# “Two-birds-one-stone” colon-targeted nanomedicine treats ulcerative colitis via remodeling immune microenvironment and anti-fibrosis

**DOI:** 10.1186/s12951-022-01598-0

**Published:** 2022-08-30

**Authors:** Jiaxin Zhang, Ante Ou, Xueping Tang, Rong Wang, Yujuan Fan, Yuefei Fang, Yuge Zhao, Pengfei Zhao, Dongying Chen, Bing Wang, Yongzhuo Huang

**Affiliations:** 1 School of Pharmacy, Institute of Interdisciplinary Integrative Medicine Research, Shanghai University of Traditional Chinese Medicine, Shanghai, 201203 China; 2 State Key Laboratory of Drug Research, Shanghai Institute of Materia Medica, Chinese Academy of Sciences, 501 Haike Rd, Shanghai, 201203 China; 3 University of Chinese Academy of Sciences, Beijing, 100049 China; 4 Artemisinin Research Center, Guangzhou University of Chinese Medicine, Guangzhou, 501450 China; 5 School of Chinese Materia Medica, Nanjing University of Chinese Medicine, Nanjing, 210023 China; 6 Laboratory of Pharmaceutical Analysis, Shanghai Institute of Materia Medica, Chinese Academy of Sciences, Shanghai, 201203 China; 7 Zhongshan Institute for Drug Discovery, SIMM, CAS, Zhongshan, 528437 China; 8 NMPA Key Laboratory for Quality Research and Evaluation of Pharmaceutical Excipients, Shanghai, 201203 China

**Keywords:** Ulcerative colitis, Colonic drug delivery, Patchouli alcohol, Simvastatin, Inflammatory microenvironment, Anti-fibrosis

## Abstract

Dysregulated mucosal immune responses and colonic fibrosis impose two formidable challenges for ulcerative colitis treatment. It indicates that monotherapy could not sufficiently deal with this complicated disease and combination therapy may provide a potential solution. A chitosan-modified poly(lactic-co-glycolic acid) nanoparticle (CS-PLGA NP) system was developed for co-delivering patchouli alcohol and simvastatin to the inflamed colonic epithelium to alleviate the symptoms of ulcerative colitis via remodeling immune microenvironment and anti-fibrosis, a so-called “two-birds-one-stone” nanotherapeutic strategy. The bioadhesive nanomedicine enhanced the intestinal epithelial cell uptake efficiency and improved the drug stability in the gastrointestinal tract. The nanomedicine effectively regulated the Akt/MAPK/NF-κB pathway and reshaped the immune microenvironment through repolarizing M2Φ, promoting regulatory T cells and G-MDSC, suppressing neutrophil and inflammatory monocyte infiltration, as well as inhibiting dendritic cell maturation. Additionally, the nanomedicine alleviated colonic fibrosis. Our work elucidates that the colon-targeted codelivery for combination therapy is promising for ulcerative colitis treatment and to address the unmet medical need.

**Graphical Abstract:**

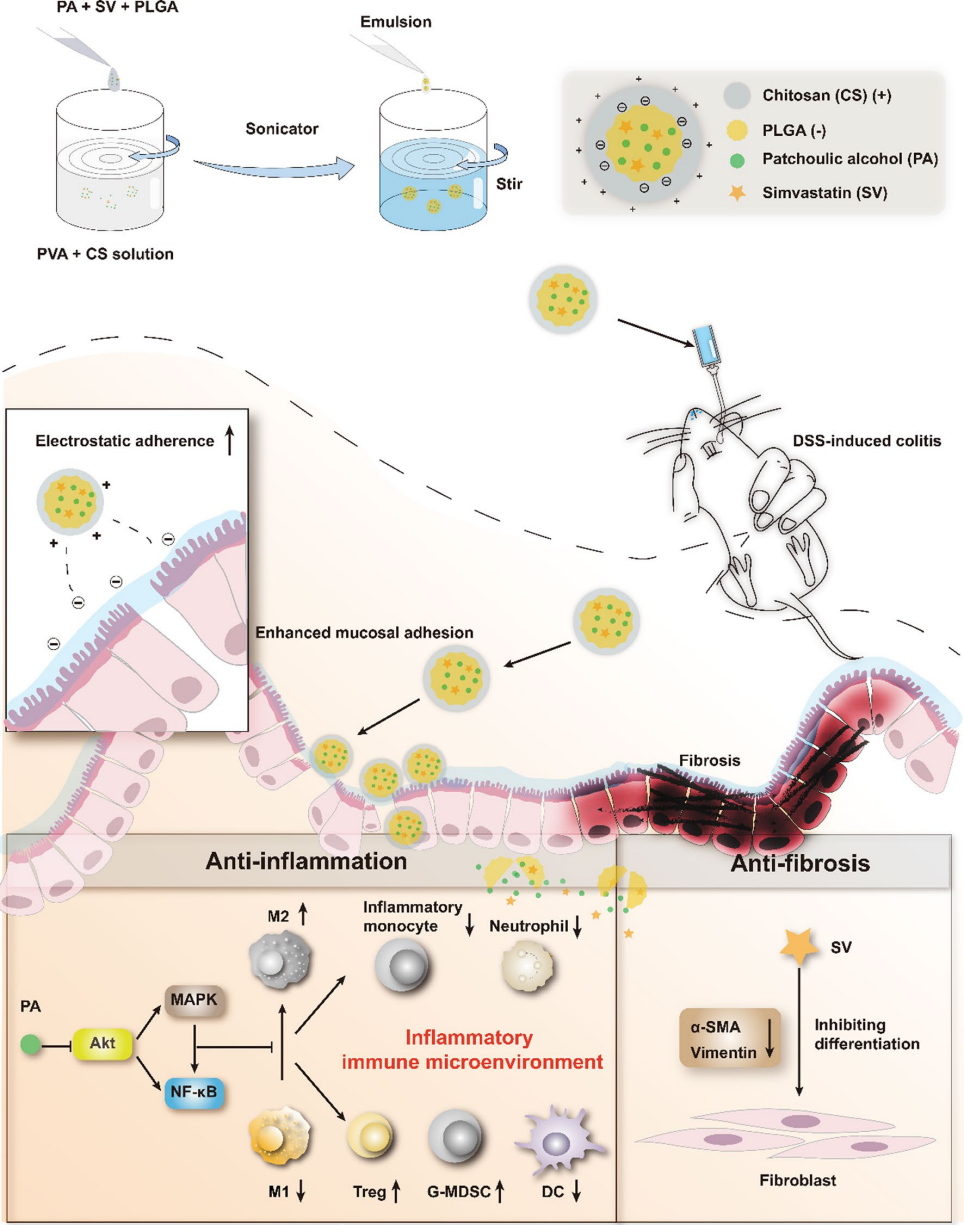

**Supplementary Information:**

The online version contains supplementary material available at 10.1186/s12951-022-01598-0.

## Introduction

Ulcerative colitis (UC) is the major form of inflammatory bowel disease (IBD). UC is a relapsing and remitting mucosal inflammation that starts from the rectum and spreads continuously to the proximal segments of the colon [1]. The prevalence of UC has been rising in the newly industrialized countries in Africa, Asia, and South America [2]. For example, it is about 11.6 per 100,000 people in China [3]. Additionally, the incidence of colorectal cancer in Asian patients with UC has been also increasing [4]. This situation imposes a great need for effective UC treatment, but the current therapy methods cannot meet the expectations due to the unsustainable efficacy [5].

The maladjustment of the immune system affected by heredity, environment, and gut microbiota is closely related to the progress of UC [6]. The intestinal immune microenvironment consists of intestinal epithelial cells, macrophages, dendritic cells (DCs), regulatory T cells (Tregs), and inflammatory T cells, which collaboratively maintain immune homeostasis [7]. The inflammatory microenvironment can be a target for UC treatment. Macrophages (MΦ) are pivotal in coordinating the progress of UC [8]. Macrophages are characterized by their diversity and plasticity in response to environmental signals and are traditionally classified into M1Φ with pro-inflammatory/anti-microbial activity and M2Φ with anti-inflammatory activity/tissue repair [9]. An increase of M1Φ amount in the pathological site of colitis predicts the worsening disease stage [10]. Re-education from M1 to M2 phenotype is a potential strategy for UC treatment [11].

In addition to the aggravated inflammatory immune responses in colitis, excessive proliferation of fibroblasts and myofibroblasts contributes to the deposition of extracellular matrix (ECM) and the fibrosis of the intestinal wall. Severe intestinal fibrosis may result in intestinal obstruction and require surgical intervention [12]. Traditional treatments mainly focus on alleviating the symptoms of UC through anti-inflammatory approaches (e.g., 5-aminosalicylic acid, corticosteroids, immunosuppressants, or monoclonal antibodies), but their clinical application has been restrained because of unsustainable therapeutic effect, the recurrence after drug withdrawal, and off-target systemic side effects [1]. Moreover, these medications are of little help in solving the intestinal fibrosis problem that is a complication of UC [12]. Therefore, the synergy of immune regulation and anti-fibrosis may be a new strategy for UC treatment.

To address this issue, we proposed a combination therapy strategy using an oral nanomedicine for co-delivering patchouli alcohol (PA) and simvastatin (SV), a “two-birds-one-stone” nanotherapeutic strategy. We previously revealed that patchouli alcohol, a natural tricyclic sesquiterpene isolated from a Chinese herb *Guang Huo Xiang* (*Pogostemon cablin* (*Blanco*) Benth.), had treatment effectiveness in alleviating colitis by remodeling the immune microenvironment [13]. Simvastatin is a common cholesterol-lowering drug. Recent studies have shown that SV-based treatment could alleviate colitis [14] and fibrosis [15, 16]. It is thus hypothesized that the combination therapy of PA and SV could yield a synergistic effect in the management of UC by targeting both the colonic immune microenvironment and fibrosis.

An oral colon-targeted drug delivery system is a promising treatment strategy for colon diseases [17, 18]. In this study, a chitosan (CS)-modified poly (lactic-co-glycolic acid) (PLGA) nanoparticulate system was developed to selectively deliver PA and SV to the colon through oral administration. Chitosan is a natural amino-polysaccharide that is easily degraded by specific enzymes produced by the colonic microflora [19], and its mucoadhesive property serves the purpose of colon-targeted delivery [20].

In this work, the developed nanomedicine was investigated for the anti-inflammatory and anti-fibrotic effects by both in vitro and in vivo experiments.

## Materials and methods

### Materials

PA was obtained from Manster Biotechnology Co., Ltd. (Chengdu, China). SV was obtained from Melone Pharmaceutical (Dalian, China). PLGA (5–15 kDa) was from Daigang Biomaterial Co., Ltd. (Jinan, China). Chitosan (< 25 kDa, degree of deacetylation 85–90%) was obtained from Yunzhou Biochemistry Co., Ltd. (Qingdao, China). Artificial colon solutions were supplied by LABEST Biotechnology Co., Ltd. (Beijing, China). Polyvinyl alcohol (PVA) and sodium carboxymethyl cellulose (CMC-Na) were obtained from Sinopharm Chemical Reagent Co., Ltd. (Shanghai, China). DiR (1,1-dioctadecyl-3,3,3,3-tetramethylindotricarbocyaine iodide) was purchased from AmyJet Scientific (Wuhan, China). Dulbecco’s modified Eagle’s medium (DMEM), fetal bovine serum (FBS), and RPMI-1640 cell culture medium were obtained from Thermo Fisher Scientific, Gibco (Waltham, USA). Macrophage colony-stimulating factor (M-CSF) and murine interleukin-4 (IL-4) were purchased from Peprotech (Rocky Hill, USA). Recombinant mouse/rat TGF-β1 was purchased from Novoprotein Co., Ltd. (Shanghai, China). 3-(4,5-Dimethyl-2-thiazolyl)-2,5-diphenyl-2-*H*-tetrazolium bromide (MTT), cocktail protease inhibitor, lipopolysaccharide (LPS), and fluorescein isothiocyanate (FITC)-dextran (average MW 3000–5000) were obtained from Sigma-Aldrich (St. Louis, USA). BCA protein assay kit, reactive oxygen species (ROS) assay kit, radioimmunoprecipitation assay (RIPA) lysis buffer, and phosphatase inhibitor cocktail A were purchased from Beyotime Biotechnology (Shanghai, China). The dextran sodium sulfate (DSS), TRIeasy™ Total RNA Extraction Reagent, RNA reverse transcription kit, and SYBR® Green Master Mix were purchased from Yeasen Biotechnology Co., Ltd. (Shanghai, China). The primary antibody of β-actin was from Sigma-Aldrich (St. Louis, USA). The primary antibody of GAPDH was from Proteintech (Rosemont, USA). The primary antibodies of phospho-NF-κB p65 (Ser536), phosphor-SAPK/JNK (Thr183/Tyr185), phospho-Akt (Ser473), Akt, p38 MAPK, Cox2, Claudin-1, and ZO-1 were purchased from Cell Signaling Technology (Boston, USA). The antibody Anti-ERK1/2, JNK/SAPK, phospho-ERK1 (Thr202/Tyr204)/ERK2 (Thr185/Tyr187), and phospho-p38 MAPK (Thr180/Tyr182) were purchased from Beyotime Biotechnology (Shanghai, China). Anti-iNOS was purchased from Absin Biological Technology Co., Ltd. (Shanghai, China). Anti-Vimentin and anti-MR antibodies were purchased from Abcam (UK). Anti-α-SMA antibody was purchased from Arigo Biolaboratories Co., Ltd. (Shanghai, China). Intracellular staining kits were purchased from BD Biosciences (Franklin Lakes, USA). APC-Cy7 anti-Mouse CD45, PE-Cy7 anti-Mouse CD11c, PE anti-Mouse MHCII, BB700 anti-Mouse CD11b, AF700 anti-Mouse Ly6C, BV605 anti-Mouse Ly6G, Percp-cy5.5 anti-Mouse CD3, and FITC anti-Mouse CD4 were purchased from BD Biosciences (Franklin Lakes, USA). FITC anti-Mouse CD11b, BV510 anti-Mouse F4/80, APC anti-Mouse CD206, FITC anti-Mouse CD45, BV421 anti-Mouse CD25, and PE anti-Mouse FoxP3 were purchased from Biolegend (USA).

### Cell lines

Human colonic carcinoma cells (Caco-2, epithelial properties), murine fibroblasts (L929), and murine macrophages (RAW 264.7) were provided by the Shanghai Cell Bank of the Chinese Academy of Sciences (Shanghai, China). The cells were cultured in DMEM (RAW 264.7, L929) and RPMI-1640 (Caco-2) media supplemented with 10% FBS and streptomycin-penicillin (100 U/mL) at 37 °C in a humidified incubator containing 5% CO_2_.

### Animals

Female or male Balb/c mice (5–7 weeks old) were purchased from the Shanghai Laboratory Animal Center (SLAC) Co., Ltd. (Shanghai, China), and housed at a specific pathogen-free care facility under a 12 h light–dark cycle. All the animal experimental procedures were complied with the institutional ethical guidelines and approved by the Institutional Animal Care and Use Committee (IACUC), Shanghai Institute of Materia Medica, Chinese Academy of Sciences (IACUC No. SYXK2015-0027).

### Mouse peritoneal macrophages isolation and polarization

Mouse peritoneal macrophages were extracted from the abdominal cavity of the male Balb/c mice injected with 2.5 mL of 3% thioglycollate medium [21]. The mice were sacrificed 4 days post-injection, and the abdominal cavity was flushed with cold PBS (10 mL). The flush-out suspension was centrifuged at 2500 rpm for 5 min to collect peritoneal macrophages. The cells were cultured in RPMI 1640 supplemented with 10% (v/v) FBS and streptomycin-penicillin (100 U/mL) for 1 day. The adherent macrophages were then exposed to LPS (1 µg/mL) to induce the M1 phenotype (M1Φ) or to IL-4 (40 ng/mL) to induce the M2 phenotype (M2Φ).

### Mouse bone marrow-derived macrophage (BMDM) culture and polarization

BMDMs were generated by culturing the bone marrow cells from the femurs and tibias of the male Balb/c mice using a previously reported protocol [22]. The cells were cultured in DMEM supplied with 20% FBS, streptomycin-penicillin (100 U/mL), and 20 ng/mL M-CSF for 4 days. The adherent macrophages were then exposed to LPS (1 µg/mL) to induce M1Φ or to IL-4 (40 ng/mL) to induce M2Φ.

### In vitro anti-fibrosis study of SV

The L929 fibroblasts were activated by transforming growth factor-β (TGF-β) (15 ng/mL). Evaluation of the anti-fibrosis effect of SV was performed in the TGF-β1-induced L929 cells. The L929 cells were co-treated with SV (1, 2, and 5 μM) and TGF-β1 (15 ng/mL) for 48 h. The cells were subjected to Western blot analysis.

### In vitro anti-inflammatory study of PA and SV and synergistic effect

The peritoneal macrophages and RAW 264.7 macrophages were pre-treated with PA (2, 5, and 10 μM) or SV (2 μM) for 2 h, respectively, and they were then exposed to LPS for 24 h to induce inflammatory macrophages. In addition, the non-treatment and M2Φ were used as control. The cells were subjected to Western blot and real-time quantitative polymerase chain reaction (qPCR) analysis.

To further investigate the synergistic effect of PA and SV, the peritoneal macrophages were pre-treated with SV (1 μM) and PA (0, 5, 10, and 20 μM) or PA (10 μM) and SV (0, 1, and 2 μM) for 2 h, respectively, and they were then exposed to LPS for 24 h to induce inflammatory peritoneal macrophages (IPM). The cells were subjected to qPCR analysis.

### Western blot analysis

The cells or tissues were lysed on ice using a RIPA lysate kit with protease and phosphatase inhibitors, and the total protein was measured by using a BCA protein assay kit. The protein samples were separated by SDS-PAGE and then transferred to polyvinylidene fluoride membrane (Millipore Merck, USA). The bands were blocked with a protein-free fast blocking solution for 10 min and incubated in the primary antibody solution prepared with 5% bovine serum albumin (BSA) overnight at 4 °C. After thorough washing with Tris-buffered saline containing 0.1% Tween-20 (TBST), the membrane was incubated with the HRP-labeled secondary antibody solution for 1 h. The membrane was washed and the proteins on the membrane were visualized using the basic luminol chemiluminescent kit (Sharebio, China) and the ChemiDoc MPTM Imaging System (Bio-Rad, USA).

### qPCR analysis

Total RNA in the cells and tissues was extracted with TRIeasy™ Total RNA Extraction Reagent, and the reverse transcription was performed with the cDNA synthesis Kit (Yeasen Biotech, China). Finally, the mixture of primers, cDNA, and SYBR was subjected to the CFX384 Touch Detecting System (Bio-Red, USA) for qPCR. All primer sequences are listed in Additional file 1: Table S1.

### Preparation of chitosan-modified PLGA nanoparticles

The PLGA nanoparticles (PLGA NPs) and chitosan-modified PLGA nanoparticles (CS-PLGA NPs) were prepared by a single emulsification-solvent evaporation method according to a previous report [23]. Briefly, 50 mg of PLGA, 5 mg of PA, and 1 mg of SV (PA/SV molar ratio around 10:1) were co-dissolved in 0.4 mL dichloromethane, and the organic solvent was added to 5 mL of PVA (1%, w/v) or PVA (1%, w/v)/chitosan (0.1%, w/v) solution using an ultrasound probe (Scientz, Ningbo, China) for 5 min under 200 W of amplitude and ice bath conditions. The obtained emulsion was added dropwise to 50 mL of PVA (0.1%) or PVA (0.1%, w/v)/chitosan (0.01%, w/v) solution under magnetic stirring condition, and then the organic solvent in the emulsion was evaporated by a rotary evaporator. The above NPs were centrifuged at 1500 rpm for 10 min to remove the unencapsulated drugs. The supernatant was collected and then centrifuged at 4 °C at 12,000 rpm. The PLGA NPs and CS-PLGA NPs were collected by washing with deionized water thrice and resuspended.

### Characterization of the CS-PLGA NPs

The particle size, polydispersity index (PDI), and potential of PLGA NPs and CS-PLGA NPs were measured by a Malvern Zeta analyzer (Nano-ZS90, Malvern, UK). The morphology of the NPs was characterized by a transmission electron microscope (TEM, 120 kV, Talos L120C, FEI, USA).

The X-ray diffractometer (XRD) spectra of the physical mixture of PA and SV, pure SV, and CS-PLGA NPs were scanned on a polycrystalline XRD (D8 ADVANCE, Bruker, Germany) at an angle in the range of 10° to 80° (2*θ*).

The drug-loading capacity (DL) and encapsulation efficiency (EE) of PA and SV in the NPs were determined by a gas chromatography–flame ionization detector (GC–FID, 6890N, Agilent Technologies, USA) and high-performance liquid chromatography (HPLC, 1260 Infinity, Agilent Technologies, USA), respectively. The calculation formula was as follows:

$${\text{DL}}\left( \% \right) = {\text{Weight}}\;{\text{of}}\;{\text{encapsulated}}\;{\text{drug}}/{\text{Total}}\;{\text{weight}}\;{\text{of}}\;{\text{nanoparticles}} \times 100\%$$

$${\text{EE}}\left( \% \right) = {\text{Weight}}\;{\text{of}}\;{\text{encapsulated}}\;{\text{drug}}/{\text{Total}}\;{\text{weight}}\;{\text{of}}\;{\text{added}}\;{\text{drug}} \times 100\%$$

The chromatographic method for detecting the concentration of PA by GC–FID is as follows. A DB-5 column (30 m × 0.25 mm × 0.25 μm) with a stationary phase of 5% phenyl–95% methyl polysiloxane was used. The temperature of the injector and detector was set to 290 °C. The heating program was kept at the starting temperature of 180 °C for 10 min. It was then raised to 290 °C (30 °C/min) and maintained at this temperature for 2 min. The split ratio was set to 20:1 and the injection volume was 2 μL.

The chromatographic method for detecting the concentration of SV by HPLC is as follows. The C18 column (250 × 4.6 mm, 5 µm, Agilent, USA) was eluted with a mobile phase composed of aqueous solution (containing 0.1% phosphoric acid, v/v) and acetonitrile (containing 0.1% phosphoric acid, v/v) (75:25, v/v) at a flow rate of 1.0 mL/min. The detection wavelength was 238 nm.

### Drug stability and in vitro drug release

SV is not stable in weakly alkaline and the SV drug stability in the NPs was measured by detecting in a simulated colonic fluid (PBS, pH 7.8). The samples were placed on a shaker at 37 °C and collected at pre-set time points for SV determination by HPLC.

The dialysis method was used to analyze the in vitro release of the PLGA NPs and CS-PLGA NPs. To simulate the in vivo drug release characteristics of the NPs, a three-stage method involving three release media with different pH values according to the Chinese Pharmacopoeia was employed. Dialysis tubes (MWCO 8–14 kDa) containing SV, PLGA NPs, or CS-PLGA NPs, respectively, were placed in the release media in an order of simulated gastric fluid (HCl, pH 1.2), simulated intestinal fluid (PBS, pH 6.8), and simulated colonic fluid (PBS, pH 7.4) containing 0.5% Tween-80. This experiment was carried out on a shaker. At the scheduled time points, the medium samples were collected and the same volume of fresh medium was replenished. The cumulative release of SV was determined by HPLC.

### Cellular uptake efficiency

The Caco-2 cells, L929 cells, and M1Φ were incubated with the coumarin 6-labeled PLGA NPs or CS-PLGA NPs for 1 h, respectively. The cells were harvested and fixed with 4% paraformaldehyde for 15 min and stained with DAPI for fluorescence imaging (Carl Zeiss, Oberkochen, Germany). The harvested cells were also analyzed by flow cytometry (ACEA NovoCyte 3000, Agilent, USA) for intracellular uptake efficiency.

### Cell viability assay

The effect of the NPs on the viability of macrophages, M2Φ, fibroblasts, and epithelial cells was determined by a standard MTT assay. The cells were treated with PA (0–80 μM), SV (0–20 μM), free combination drugs, PLGA NPs, and CS-PLGA NPs (molar ratio around 10:1) for 24 h, respectively. The microplate reader (Multiskan, Thermo Fisher, USA) was used to measure the optical density value of the sample at 490 nm.

### Measurement of intracellular ROS

The BMDM cells were pretreated with free drug combination, PLGA NPs, or CS-PLGA NPs (molar ratio around 10:1) for 2 h and then incubated with LPS (1 μg/mL) for 6 h, respectively. The cells were collected for determining ROS by using a ROS determination kit and flow cytometry.

### Measurement of intracellular inflammatory cytokines

The RAW 264.7 cells were pretreated with free drug combination (PA 10 μM + SV 1 μM), PLGA NPs, or CS-PLGA NPs (equal dose to the combination) for 2 h and then incubated with LPS (1 μg/mL) for 24 h. These cells were subjected to qPCR assay to detect the mRNA expression of inflammatory cytokines.

### In vivo distribution of nanoparticles

To verify the targeting ability of CS-PLGA NPs to inflammatory sites, IVIS was employed. Briefly, the colitis mice were fasted overnight before the beginning of the experiment, and then orally administered with DiR-labeled PLGA NPs or CS-PLGA NPs. The mice were sacrificed and the organs (heart, liver, spleen, lung, kidney, intestine, and colon) were dissected for ex vivo imaging at the predetermined time points (3 and 5 h), and then the organs were exposed to the IVIS imaging system (Caliper PerkinElmer, Hopkinton, USA) to analyze the biodistribution of the NPs.

### In vivo treatment of acute colitis

The therapeutic effect of CS-PLGA NPs was evaluated using an acute colitis model, which was induced in the Balb/c mice by supplementing with 3% (w/v) DSS-containing water for 13 days. The colitis mice were randomly allocated into four treatment groups (DSS, PA/SV, PLGA NPs, and CS-PLGA NPs), while a healthy group was set up as a control. The free drugs dispersed in 0.5% CMC-Na (w/v) aqueous solution or the NPs were orally administered according to a regimen in Fig. 4A at a dose of 16 mg/kg of PA and 3.2 mg/kg of SV (i.e., 10:1 mol/mol) (n = 6 per group). During the experiment, the body weight, feces, and disease activity index (DAI) of the mice were recorded daily. The DAI score was calculated as shown in Additional file 1: Table S2. At the end of the experiment, the mice were sacrificed, the colon was separated and measured for length, and the main organs were collected and weighed to calculate the organ coefficient according to the following formula:

$${\text{Organ}}\;{\text{coefficient}}\left( \% \right) = {\text{Weight}}\;{\text{of}}\;{\text{organ}}/{\text{Weight}}\;{\text{of}}\;{\text{mice}}$$

The colon, heart, liver, spleen, lung, and kidney were collected and fixed with 4% paraformaldehyde and stained with hematoxylin/eosin (H&E) for pathological analysis. In addition, the colon tissue was subjected to Masson’s trichrome staining and immunohistochemistry assay.

### In vivo analysis of intestinal permeability

The FITC-dextran was used to measure epithelial permeability, as described previously [13]. Briefly, the mice were starved overnight before the end of the experiment. The mice were treated with FITC-dextran (220 mg/kg) orally. After 4 h, the blood samples were collected. The fluorescence intensity in the serum was measured in a microplate reader at an excitation wavelength of 485 nm and an emission wavelength of 528 nm.

### Flow cytometry analysis

The colon tissues were harvested for flow cytometry analysis according to a previously used method [24]. The colon tissues were incubated with a digestive solution of intestinal epithelial cells (HBSS, 1 mM EDTA, and 1 mM DTT) to remove intestinal epithelial cells, and then enzymatically digested in RPMI 1640 media containing collagenase IV (1 mg/mL), DNase I (0.3 mg/mL), and 5% FBS at 37 °C. The colonic lamina propria cells were harvested by centrifugation and blocked with PBS containing 2% BSA. M2Φ and DCs were labeled with CD45-APC-Cy7, CD11b-FITC, CD11c-PE-Cy7, MHCII-PE, F4/80-BV510, and CD206-APC antibodies. The pro-inflammatory monocytes, neutrophils, and G-MDSCs were labeled with CD45-FITC, CD11b-BB700, Ly6C-AF700, and Ly6G-BV605 antibodies. Tregs were labeled with CD45-APC-Cy7, CD3-Percp-cy5.5, CD4-FITC, and CD25-BV421. Intracellular FoxP3-PE was stained using an intracellular staining kit (BD Biosciences, USA). The cells were measured with a flow cytometer (ACEA NovoCyte 3000, Agilent, USA).

### Statistical methods

All data were expressed as mean ± SD (n ≥ 3). Statistical analysis was performed by Student’s t-test or one-way ANOVA. Statistical significance was indicated as *P < 0.05, **P < 0.01, and ***P < 0.001.

## Results and discussion

### Effect of SV on fibroblast activation

**Fig. 1:**
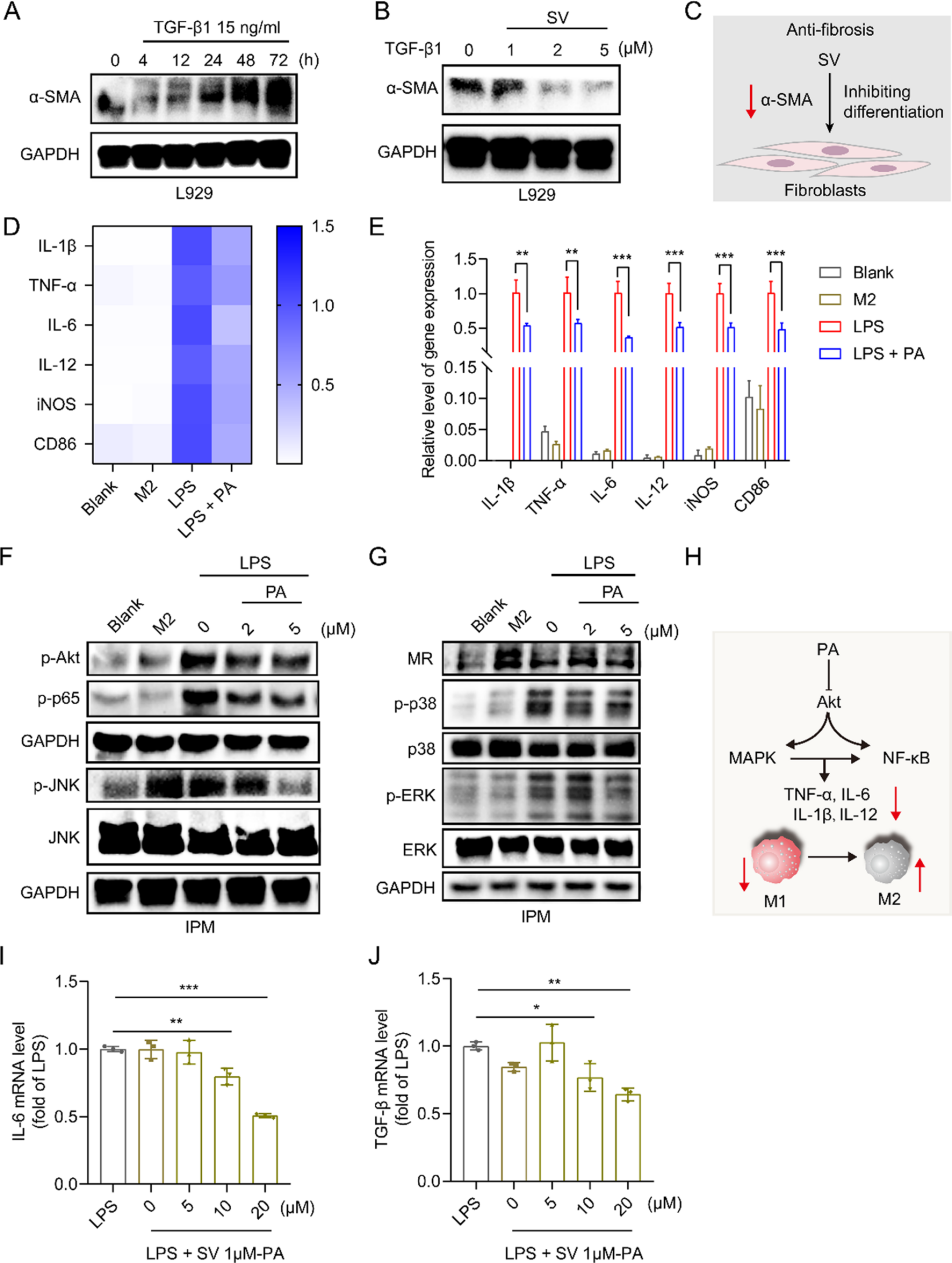
SV-induced anti-fibrosis, PA-mediated macrophage repolarization, and synergistic effect of the drug combination. **A** Western blot analysis of fibroblast activation-associated α-SMA in L929 cells induced by TGF-β with varying exposure duration. **B** The inhibition of fibroblast activation after SV treatment. **C** Scheme of SV-induced anti-fibrosis. **D** The heatmap and **E** mRNA levels of M1-associated pro-inflammatory cytokines (e.g., IL-6, IL-1β, TNF-α, and IL-12) and markers (e.g., CD86 and iNOS) in PA-treated inflammatory peritoneal macrophages measured by qPCR. **F**, **G** Western blot analysis of Akt/MAPK/NF-κB pathway-related biomarker and M2-related MR expression after PA treatment. **H** Scheme of macrophage repolarization after PA treatment. **I**, **J** The downregulation of the pro-inflammatory IL-6 and fibroblast activator TGF-β after drug treatment with different combined molar ratios, as measured by q-PCR

Fibrosis is a consequence of the expansion of mesenchymal cells (including fibroblasts, myofibroblasts, and smooth muscle cells), which causes the accumulation of collagen-rich ECM [25]. TGF-β is a central regulator that activates fibroblasts and induces fibrosis [26]. The TGF-β/Smad2/3 pathway is critical to intestinal fibrosis by inducing the differentiation of fibroblasts into myofibroblasts [12]. It is reported that TGF-β1-mediated phosphorylation of Smad-3 could be reversed by the SV treatment [27]. The TGF-β1-induced L929 fibrotic cell model was established. Figure 1A shows the TGF-β1-induced fibroblast activation in a time-dependent manner, as evidenced by the upregulation of α-Smooth muscle actin (α-SMA), a marker for activated fibrogenic cells. The activation of fibroblasts induced by TGF-β1 was successfully reversed by SV treatment (Fig. 1B, C), indicating the anti-fibrosis effect of SV.

### Effect of PA on macrophage repolarization and synergistic effect with SV

Macrophages are the major mediator in colitis [8]. The macrophage repolarization effect of PA from M1 to M2 phenotype was demonstrated by the decreased mRNA levels of the M1-related pro-inflammatory cytokines (e.g., IL-6, IL-1β, TNF-α, and IL-12) and markers (e.g., iNOS and CD86) (Fig. 1D, E, Additional file 1: Fig. S1A–C), whereas the M2-related mannose receptor (MR) was upregulated (Fig. 1G, Additional file 1: Fig. S1D).

The phosphatidylinositol-3 kinases/protein kinase B (PI3K/Akt) pathway is widely involved in inflammatory diseases [28, 29]; PI3K can activate Akt that subsequently relocates to the cell membrane [30], thereby modulating mitogen-activated protein kinases (MAPKs) and nuclear factor-кB (NF-κB) signaling pathways [31, 32]. MAPKs/NF-κB signaling pathway is essential for M1Φ polarization [33]. MAPKs include extracellular signal-regulated kinase (ERK1/2), c-Jun NH2 terminal kinase (JNK), p38, and ERK5. Activated NF-κB unregulates inducible nitric oxide synthase (iNOS), cyclooxygenase-2 (COX-2), IL-1β, and tumor necrosis factor α (TNF-α) [34, 35]. The Akt/MAPK/NF-κB signaling pathway in the M1Φ was examined after treatment with PA. It was found that the phosphorylation levels of Akt, ERK, JNK, p38, and NF-κΒ p65 were up-regulated in the LPS-treated inflammatory macrophages (M1 phenotype), but this effect was reversed by PA treatment (Fig. 1F, G, Additional file 1: Fig. S1D, E). The results suggested that PA could repolarize the M1Φ and suppress the inflammatory responses through the mechanism of the Akt/MAPK/NF-κB signaling pathway (Fig. 1H). These results were consistent with the findings of our previous work [13].

We further investigated the effect of SV on the macrophages. The results showed that SV had no significant effect on the M1-related pro-inflammatory molecules (e.g., TNF-α, iNOS, and COX-2), but upregulated the M2-related Arg1 (Additional file 1: Fig. S1F). It indicated that SV might have a minor effect on macrophages, but not play a major role in this case at the selected dose. However, SV in combination with PA showed synergistic inhibition of macrophages-secreted IL-6 (Additional file 1: Fig. S1G). Moreover, we further found that SV showed a bidirectional effect on macrophages; i.e., at a low dose (1 μM) it was anti-inflammatory, but at a high dose (2 μM) it was pro-inflammatory (Additional file 1: Fig. S1G). We thus fixed the concentration of SV (1 μM) to test varying concentrations of PA for optimizing the PA/SV ratio. As shown in Fig. 1I, J, the PA/SV molar ratios of both 10:1 and 20:1 exerted anti-inflammatory and anti-fibrosis synergistic effects. Therefore, the PA/SV molar ratio of 10:1 was chosen for further investigation. Importantly, PA and SV were of biosafety in the epithelial cells, macrophages, and fibroblasts (Additional file 1: Fig. S2A–C).

The synergistic effect of the combination of PA/SV was preliminarily evaluated in the DSS-induced colitis model (Additional file 1: Fig. S3A). The combination therapy effectively alleviated the colitis symptoms, reflected by the reduced weight loss and colon length shrinkage, with higher efficacy than monotherapy (Additional file 1: Fig. S3B–D). In addition, oral administration of PA and SV showed no obvious organ toxicity (Additional file 1: Fig. S4A, B). The pilot results first demonstrated that the combination therapy of an anti-inflammatory modulator and anti-fibrosis drug yielded a synergistic effect on alleviating colitis.

### Characterization of CS-PLGA NPs

**Fig. 2:**
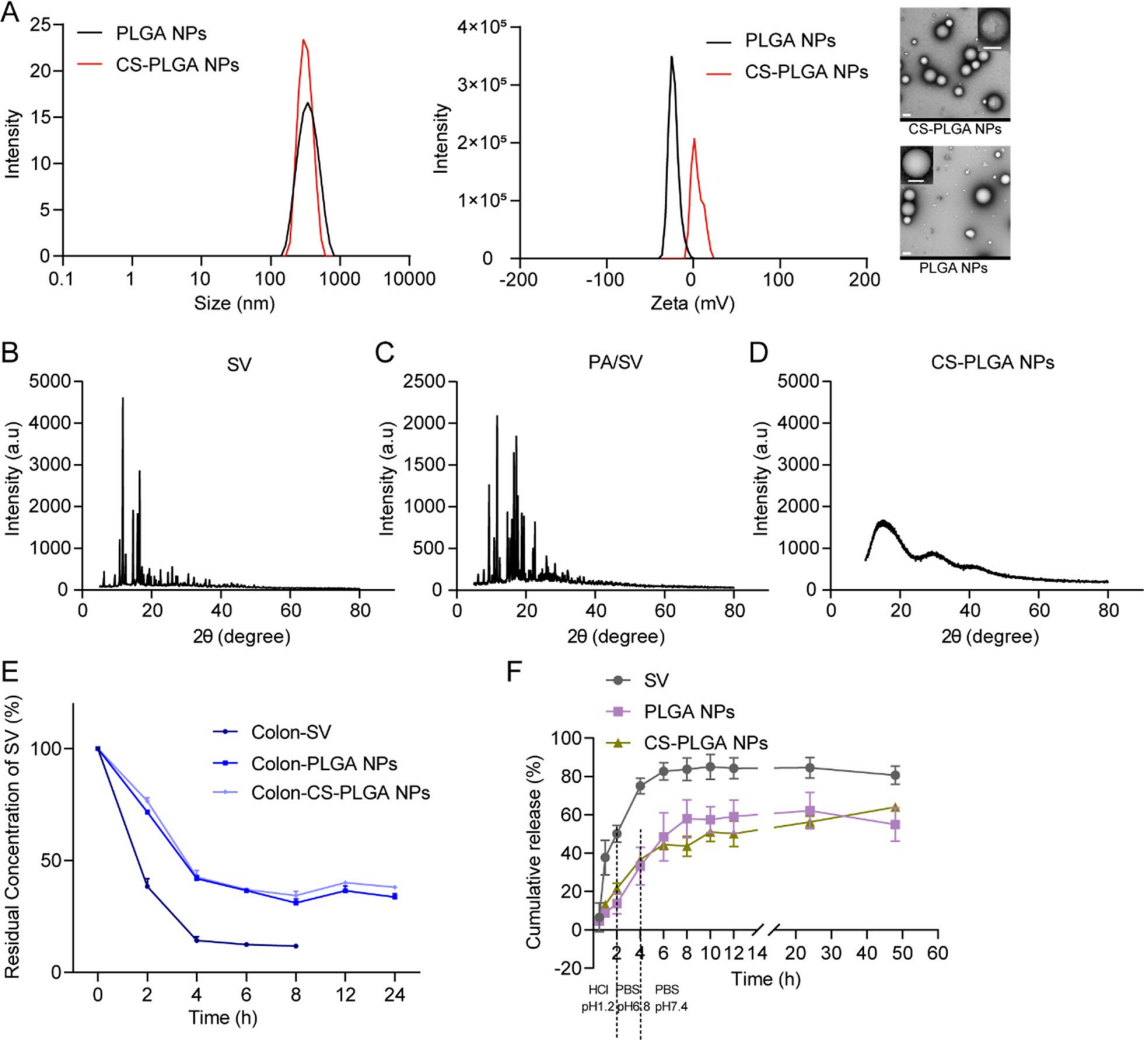
Characterization of the NPs. **A** The size distribution, ζ potential, and TEM image (scale bar: 200 nm) of PLGA NPs and CS-PLGA NPs. **B**–**D** XRD spectra. **E** Stability of the NPs in simulated colonic fluids. **F** Cumulative release of SV in three release media with different pH

The CS-PLGA NPs with co-encapsulation of PA/SV were fabricated via a single emulsification-solvent evaporation method. The size of the CS-PLGA NPs was about 350 nm (Fig. 2A, Additional file 1: Table S3). Due to the coating of chitosan, the CS-PLGA NPs were positively charged (9.7 mV) (Fig. 2A, Additional file 1: Table S3). The DL% and EE% of the CS-PLGA NPs are shown in Additional file 1: Table S4. The XRD spectrum of SV and physical mixture showed sharp peaks, whereas SV in the CS-PLGA NPs showed an amorphous form as solid dispersion (Fig. 2B–D).

### Stability and in vitro drug release of CS-PLGA NPs

The chemical stability of SV and the nanomedicines was evaluated in the simulated colonic fluid (pH 7.8). SV showed poor stability in a weak alkaline condition, while the CS-PLGA NPs could effectively protect SV against degradation and improve its stability (Fig. 2E). The in vitro release percentage of the free SV in the simulated gastric fluids and simulated intestinal fluid totaled 75%, compared to about 35% for the PLGA NPs and CS-PLGA NPs (Fig. 2F). However, in the simulated colonic fluid, drug release from CS-PLGA NPs reached 64.1%, and PLGA NPs reached 54.9%. This release behavior indicated the enhanced colonic release of oral drugs from the NPs [36].

### Cytotoxicity and cell uptake assay

**Fig. 3:**
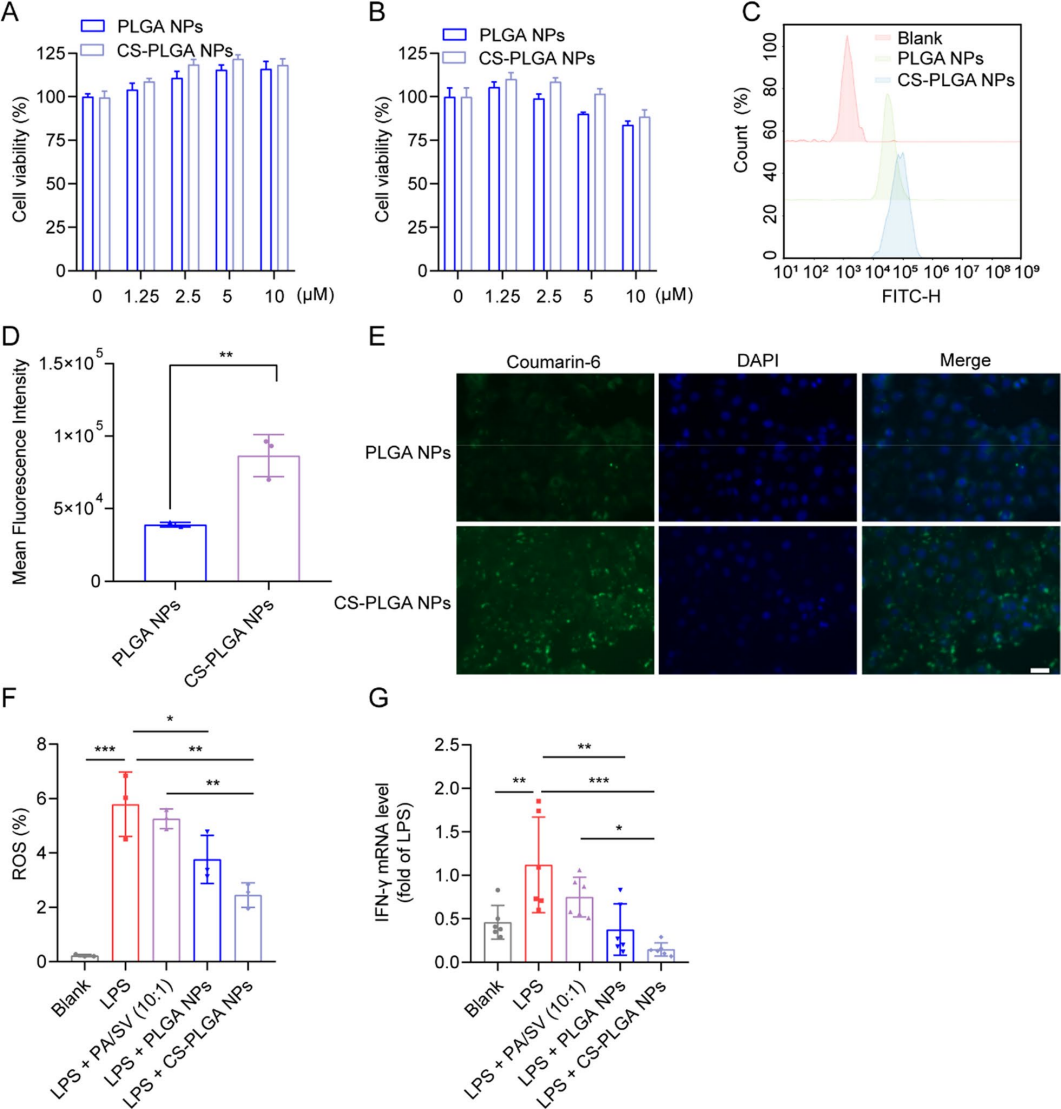
CS-PLGA NP-mediated anti-inflammatory and cellular uptake in vitro. Cytotoxicity of the NPs in Caco-2 cells (**A**) and RAW264.7 cells (**B**). Histogram (**C**) and mean fluorescence intensity (**D**) of the NP-internalized Caco-2 cells were analyzed by flow cytometry. **E** Fluorescence images of Caco-2 cells after incubation with the coumarin 6-labeled NPs (scale bar: 50 µm). **F** ROS levels were reduced in the LPS-induced RAW264.7 cells after drug treatment. **G** IFN-γ levels decreased in drug-treated M1Φ

The CS-PLGA NPs showed high biocompatibility with the epithelial cells, macrophages, and fibroblasts (Fig. 3A, B, Additional file 1: Fig. S2D, E). When the concentration of the CS-PLGA NPs was high up to 5 μM (indicated by SV), the cell viability was still larger than 80%. It also demonstrated the biosafety of chitosan for oral delivery.

Furthermore, the effect of chitosan on the uptake of NPs in a Caco-2 epithelial cell model that secretes extracellular mucus was investigated. The chitosan modification on the PLGA NPs significantly enhanced drug delivery in the epithelial cells, compared to the non-modified PLGA NPs (Fig. 3C–E). The results were in accordance with other reports of chitosan-promoted penetration of the epithelial cells [37]. Furthermore, the CS-PLGA NPs significantly enhanced uptake in the M1Φ (Additional file 1: Fig. S5A, C, D) and fibroblasts (Additional file 1: Fig. S5B, E, F) compared to the PLGA NPs.

### Anti-inflammatory and anti-oxidative stress by CS-PLGA NPs

As an oxidative mediator in the inflammatory microenvironment, ROS serves as a “trigger” that facilitates the production of pro-inflammatory cytokines (e.g., IL-1β and IL-18) in macrophages, and thus promotes the progression of colitis [38, 39]. Our results revealed that the CS-PLGA NPs suppressed ROS generation in the inflammatory macrophages (Fig. 3F). Additionally, the IFN-γ level in the inflammatory macrophages was also reduced (Fig. 3G). The results demonstrated the anti-inflammatory and anti-oxidative stress effects of CS-PLGA NPs on the inflammatory macrophages.

### In vivo distribution of the NPs

The accumulation of CS-PLGA NPs in inflamed colons was investigated in the DSS-induced colitis model. Ex vivo IVIS imaging revealed that DiR-bearing NPs primarily accumulated in inflamed intestines and colons than in other organs (Additional file 1: Fig. S6A, B, D, E). The CS-PLGA NPs exhibited an enhanced colon tissue accumulation (Additional file 1: Fig. S6C, F).

It should be mentioned that chitosan has the characteristic that quickly and reversibly opens tight junctions to facilitate drug delivery to the colonic epithelium [36]. The inflamed colon tissue is characterized by the thinning mucus layer that is caused by the decrease of goblet cells and mucin in UC patients [40]. Therefore, the chitosan nanoparticles can take advantage of the thinning mucus layer, the mucosal adhesion, and the ability to open the tight junctions in the inflammatory area.

### In vivo treatment in DSS-induced colitis

**Fig. 4:**
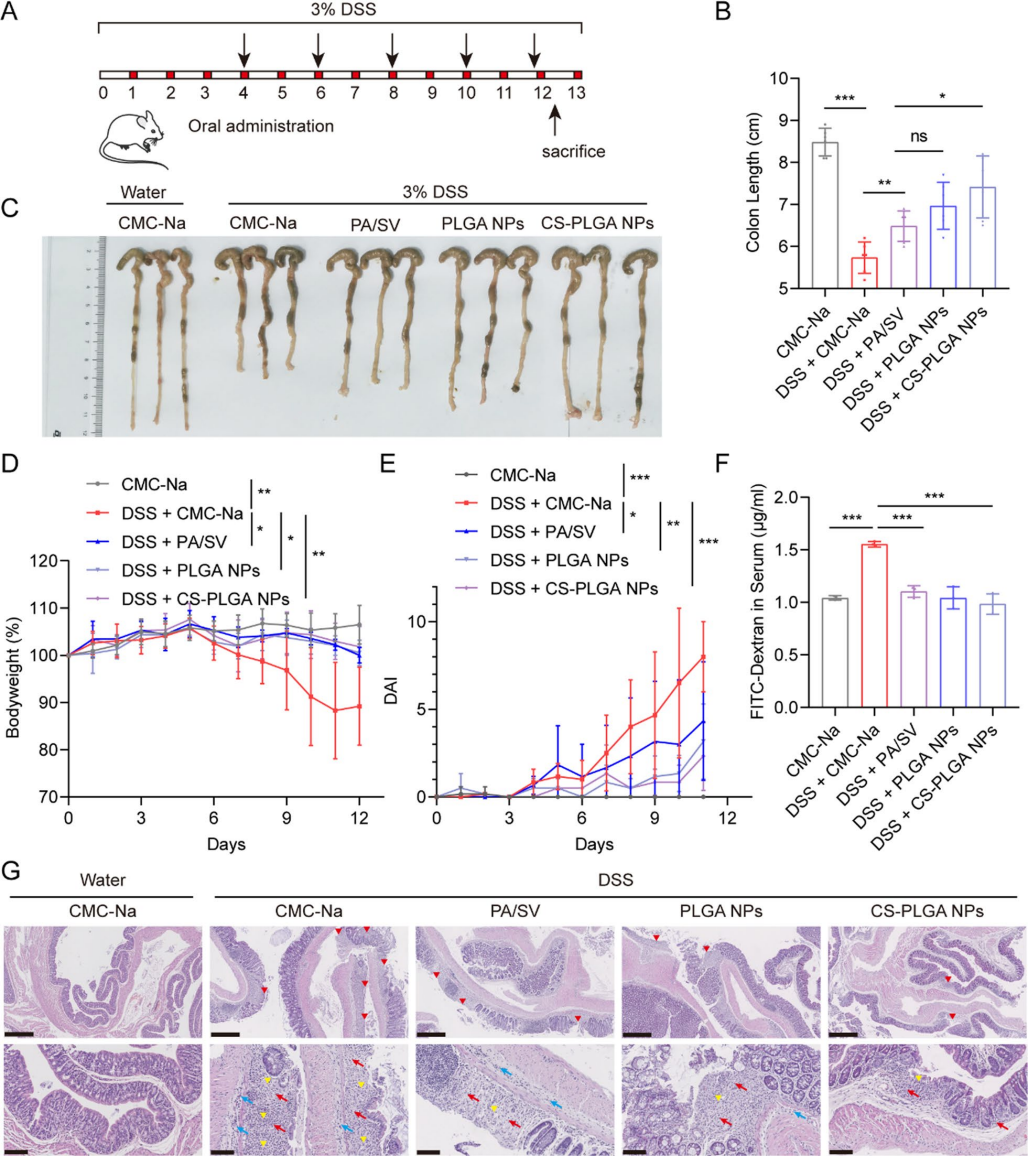
Anti-colitis treatment of the CS-PLGA NPs. **A** Schematic diagram of DSS-induced colitis and treatment regimen. **B** Drug treatment alleviated the shrinkage of colon length. **C** Images of the colon. **D** Treatment alleviated bodyweight loss. **E** DAI in the treatment period. **F** Treatment alleviated the intestinal permeation caused by colitis. **G** Histopathological assessment of colon tissue sections stained with H&E. The upper panels (scale bar: 500 μm) show the loss of surface epithelium marking ulceration (red arrowhead). The lower panels (scale bar: 100 μm) show the hyperplastic connective tissue (yellow arrowhead) resulting from ulcer lesion and inflammatory cell infiltration into mucosa (red arrow) or submucosa (blue arrow)

The therapeutic efficacy of the CS-PLGA NPs was examined in an acute colitis mouse model induced by DSS (Fig. 4A). The CS-PLGA NP treatment effectively alleviated the colitis symptoms including colon length shrinkage (Fig. 4B, C), bodyweight loss (Fig. 4D, Additional file 1: Fig. S7A–E), and the increased DAI score (Fig. 4E, Additional file 1: Fig. S7F–J). Of note, the mucosal barrier is important to maintain gut homeostasis and normal functions, and the mucosal barrier is typically impaired in UC and characterized by the increased intestinal permeability of the colon [41]. Restoring the barrier functions is a potential therapeutic method [42]. The CS-PLGA NP treatment alleviated the intestinal permeability of the colon (Fig. 4F), exhibiting better effectiveness than other groups, as reflected by the reduced serum level of the orally administered FITC-dextran that serves as an intestinal leakage indicator.

Histological examination showed that there were extensive erosions and ulcerations in the colons of the non-treated colitis mice, and the colonic epithelium was damaged, with marked inflammatory cell infiltration into the mucosa and submucosa. The combination therapy of PA/SV revealed the therapeutic efficacy against colitis. The CS-PLGA NPs displayed the best treatment outcomes (Fig. 4G), and effectively protected against colon epithelium damage and reduced inflammatory cell infiltration. In addition, there was no obvious organ side toxicity (Additional file 1: Fig. S8A, B).

It should be pointed out that treatment of CS-PLGA NPs showed improved efficacy compared to the PLGA NPs, but no statistical difference was shown between them. However, the CS-PLGA NPs did exhibit significant improvement than the non-treatment group, while the PLGA NPs not (Fig. 4D, E). The statistics could be affected by the limited sample size; there were a limited amount of animals available in our approved protocol. More investigations should be conducted to further reveal the benefits of the CS-PLGA NPs, though.

### Anti-inflammatory and anti-fibrotic mechanisms

**Fig. 5:**
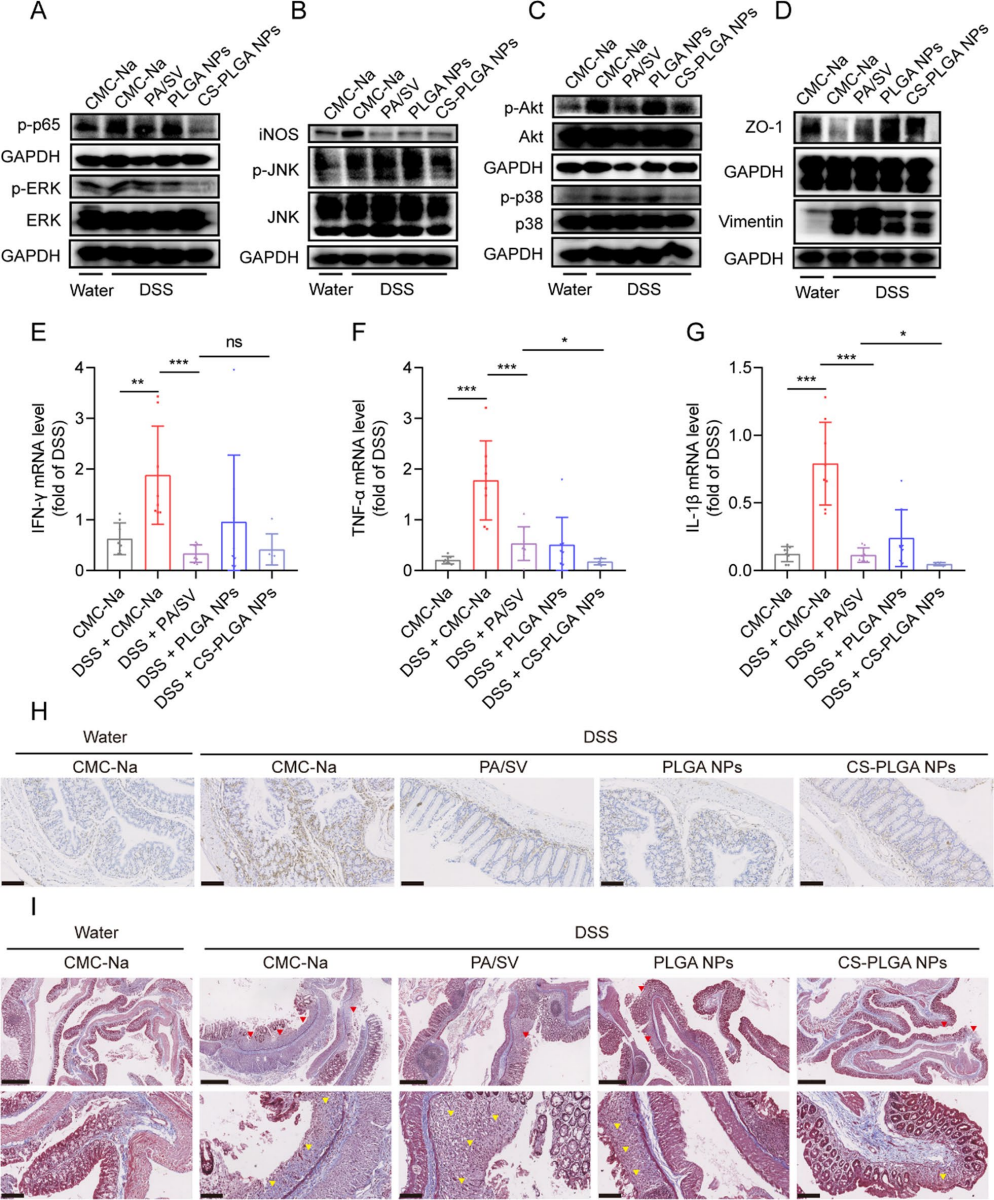
Anti-inflammatory and anti-fibrotic mechanisms. **A**–**C** Inhibition of Akt/MAPK/NF-κB pathway and downregulation of iNOS in colon tissues after treatment. **D** Upregulation of ZO-1 and downregulation of vimentin in colon tissues after treatment. **E**–**G** The colonic mRNA level of pro-inflammatory cytokines (e.g., IFN-γ, TNF-α, and IL-1β). **H** Immunohistochemical staining of vimentin (brown color) (scale bar: 100 μm). **I** Histopathological assessment of colon sections stained with Masson's trichrome. The upper panels (scale bar: 500 μm) show the loss of surface epithelium marking ulceration (red arrowhead). The lower panels (scale bar: 100 μm) show the hyperplastic connective tissues (yellow arrowhead) and the blue-colored hyperplastic collagen fibers

The PI3K/Akt/MAPK/NF-κB signaling pathway is widely involved in the progression of colitis [43]. As shown in Fig. 5A–C, the phosphorylation of Akt, JNK, ERK, p38, and p65 in the colitis group were activated. The inhibition of Akt/MAPK/NF-κB signaling was found in the group treated with the CS-PLGA NPs (Fig. 5A–C), accompanied by the down-regulation of the pro-inflammatory cytokines (e.g., IFN-γ, TNF-α, and IL-1β) (Fig. 5E–G) and M1Φ-related iNOS (Fig. 5B). The results indicated that the CS-PLGA NPs suppressed the M1Φ polarization in inflammatory colonic tissue.

IBD-associated fibrosis is manifested as excessive and abnormal ECM deposition, which leads to scar formation, tissue deformation, and intestinal obstruction [25]. UC-associated fibrosis along with muscularis mucosae thickening appears in the inflammatory area and is linked to the severity of inflammation [44]. The CS-PLGA NP treatment down-regulated the fibrosis-related vimentin, but did not affect the tight junction protein ZO-1 (Fig. 5D, H) which is an indicator of the structural integrity of the colonic epithelial barrier. In the normal colon, the collagen fibers (blue staining) were present mostly in the submucosa; by contrast, the colitis tissue was hyperplasia in the mucosa (Fig. 5I). Importantly, the CS-PLGA NP treatment effectively improved the connective tissue and collagen fiber hyperplasia in the ulcer lesion. These results confirmed that the CS-PLGA NPs displayed protective effects against colitis through anti-inflammatory and anti-fibrotic mechanisms.

Our results showed that the impaired colonic epithelial barrier, indicated by the down-regulated tight junction protein ZO-1, is accompanied by increased fibrosis-associated vimentin in colitis mice (Fig. 5D, H), suggesting fibrosis might undermine the epithelial barrier. However, to further illustrate the relationship between fibrosis and colonic permeability still needs further investigations.

### Remodeling of the inflammatory immune microenvironment

**Fig. 6:**
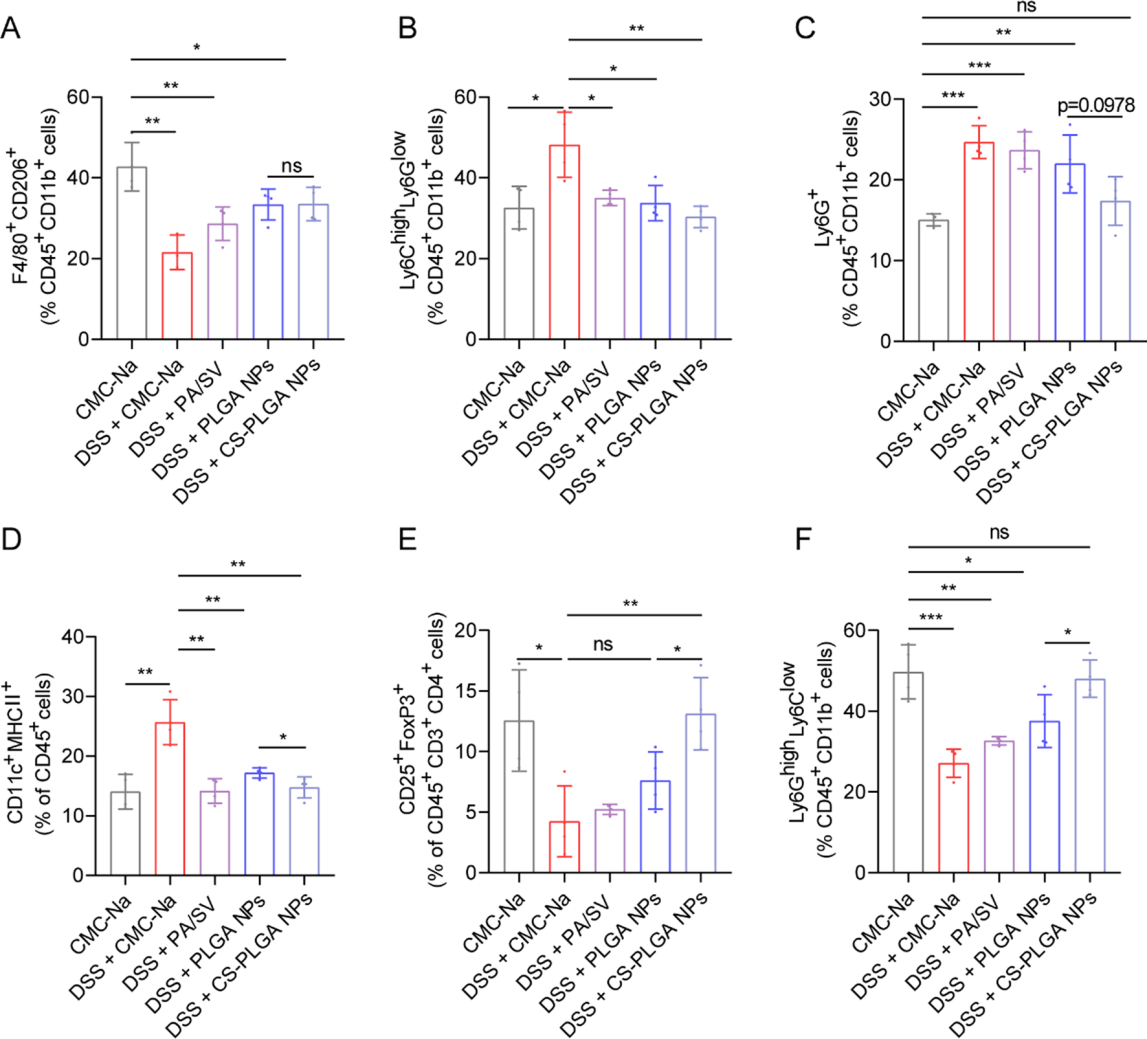
Remodeling the inflammatory immune microenvironment in the colon tissues. **A** The CS-PLGA NP-promoted M2Φ polarization (F4/80^+^ CD206^+^). **B** Inflammatory monocytes (Ly6C^high^ Ly6G^low^). **C** Colonic infiltration of neutrophils (Ly6G^+^). **D** CS-PLGA NP-induced DC maturation (CD11c^+^ MHCII^+^). **E** CS-PLGA NP-accelerated Tregs (CD25^+^ FoxP3^+^). **F** Colonic infiltration of G-MDSCs (Ly6G^high^ Ly6C^low^)

UC is characterized by the imbalance of the innate and adaptive immune responses triggered by colonic dysfunctions; the cytokines produced by the activated immune cells can cause the impairment of intestinal barrier function and trigger a perpetuated gut inflammation [7, 45]. Therefore, reprogramming the colonic immune cells and remodeling the inflammatory immune microenvironment may be a promising strategy to alleviate UC [6]. The innate responses were investigated after treatment, and the population of CD206^+^ M2Φ was increased to 33.5% in the CS-PLGA NPs group, whereas it was 21.6% in the non-treated colitis (DSS) group (Fig. 6A, Additional file 1: Fig. S9). Of note, the colonic inflammatory macrophages can trigger the recruitment of pro-inflammatory monocyte precursors [8]. Our result revealed that the CS-PLGA NP treatment significantly reduced the proportion of pro-inflammatory monocytes (Ly6G^low^ Ly6C^high^) in the colon tissue (Fig. 6B, Additional file 1: Fig. S10).

IBD is accompanied by the infiltration of a large number of neutrophils, impairment of the epithelial barrier function, and release of inflammatory mediators [46]. Additionally, ROS induces chemokine production that promotes the recruitment of inflammatory cells, and TRPM2 Ca^2+^ influx is associated with ROS-induced chemokine generation in monocytes and macrophages, thereby increasing neutrophil infiltration [47]. In accordance with the in vitro results of the potent immune-modulatory effect of the CS-PLGA NPs (Fig. 3F), the CS-PLGA NP treatment reduced the percentage of neutrophils (Ly6G^high^) in the colitis tissues (Fig. 6C, Additional file 1: Fig. S10).

Macrophages and DCs, as antigen-presenting cells, play an important role in triggering the innate and adaptive immune responses. It was found that a 1.7-fold decrease in the proportion of DCs after the CS-PLGA NP treatment (Fig. 6D, Additional file 1: Fig. S9) compared with the DSS group. Furthermore, the populations of Tregs (CD25^+^ FoxP3^+^) and granulocytic myeloid-derived suppressor cells (G-MDSC, Ly6G^high^ Ly6C^low^) were elevated to 2.6-fold and 1.8-fold, respectively, after the CS-PLGA NP treatment (Fig. 6E, F, Additional file 1: Figs. S10, S11). These results revealed that the CS-PLGA NPs re-educated M1Φ into M2Φ and reshaped the immune microenvironment in the colon tissue via suppressing the pro-inflammatory immune cells (e.g., pro-inflammatory monocytes, DCs, and neutrophils) and promoted the immunosuppressive cells (e.g., Tregs and G-MDSCs). The remodeling immune microenvironment, in turn, could alleviate colonic fibrosis.

## Conclusion

A mucoadhesive nanomedicine was developed for codelivery of PA and SV to the inflamed epithelium for a synergistic effect on anti-inflammation and anti-fibrosis. The chitosan modification enhanced the colonic drug delivery efficiency and improved the drug stability in the GI tract. This nanomedicine alleviated colitis via targeting the Akt/MAPK/NF-κB pathway, remodeled the inflammatory immune microenvironment, and inhibited fibroblast activation. This oral colon-targeted codelivery strategy and dual-action therapeutic method are promising for developing a safe and effective drug for UC. The interaction between the inflammatory immune microenvironment and colitis-related fibrosis during the progression of UC has not been fully demonstrated yet, and further investigation and understanding will be helpful to better depict the underlying mechanisms and seek effective drug combinations.

## Supplementary Information

**Additional file 1: Figure S1.** Effect of PA on macrophage repolarization and synergistic effect with SV. (A–C) The mRNA levels of M1-associated pro-inflammatory cytokines (e.g., IL-1β, IL-6, and TNF-α) in PA-treated RAW264.7 macrophages, as measured by qPCR. (D, E) Western blot analysis of Akt/MAPK/NF-κB pathway-related biomarkers and M2-related MR expression after PA treatment. (F) The mRNA levels of the M1-related pro-inflammatory molecules (e.g., TNF-α, iNOS, and COX-2) and M2-related Arg1 in SV-treated RAW264.7 macrophages, as measured by qPCR. (G) IL-6 mRNA levels in LPS-induced peritoneal macrophages treated with PA (10 μM) and SV (0, 1, and 2 μM). **Figure S2.** Cytotoxicity study of PA and SV on (A) Caco-2 cells, (B) RAW264.7 cells, and (C) L929 cells. Cytotoxicity of the NPs in (D) L929 cells and (E) M2Φ. **Figure S3.** Anti-colitis treatment of synergistic drugs. (A) Schematic diagram of DSS-induced colitis and treatment. (B) Changes in daily bodyweight of each group during the trial period. (C) Statistical analysis and (D) images of colon lengths in each group (n = 4). **Figure S4.** Preliminary biosafety assessment of PA and SV. (A) Organ coefficients. (B) H&E staining of the major organs (Scale bar: 100 μm). **Figure S5.** Fluorescence images of (A) M1Φ and (B) L929 after incubation with the coumarin 6-labeled NPs (scale bar: 50 µm). (C, E) Histogram and (D, F) mean fluorescence intensity of the NPs-internalized M1Φ (LPS-induced RAW264.7 cells) and L929 cells were analyzed by flow cytometry (n = 3). **Figure S6.** Specific accumulation of CS-PLGA NPs in inflamed colons. Ex vivo imaging and radiant efficiency of (A, B) organs and (C) colons at 3 h. Ex vivo imaging and radiant efficiency of (D, E) organs and (F) colons at 5 h (n = 3). **Figure S7.** (A–E) Individual bodyweight curves and (F–J) DAI curves in CMC-Na, DSS, PA/SV, PLGA NPs, or CS-PLGA NPs groups (n = 6). **Figure S8.** Preliminary biosafety assessment. (A) Organ coefficients. (B) H&E staining of the major organs (Scale bar: 100 μm). **Figure S9.** The dot plots of M2Φ and DCs in the colon tissue. **Figure S10.** The dot plots of neutrophils, inflammatory monocytes, and G-MDSCs in the colon tissue. **Figure S11.** The dot plots of Tregs in the colon tissue. **Table S1.** The primer sequence used in qPCR. **Table S2.** Disease activity index (DAI) scoring. **Table S3.** Characterization of the NPs. **Table S4.** Drug encapsulation efficiency and drug loading efficacy.

## Data Availability

All data generated or analyzed during this study are included in this published article and its Additional file.

## Abbreviations

MTT: 3-(4,5-Dimethyl-2-thiazolyl)-2,5-diphenyl-2-*H*-tetrazolium bromide
CS: Chitosan
JNK: C-Jun NH2 terminal kinase
COX-2: Cyclooxygenase-2
DCs: Dendritic cells
DSS: Dextran sodium sulfate
DiR: 1,1-Dioctadecyl-3,3,3,3-tetramethylindotricarbocyaine iodide
DL: Drug-loading capacity
DMEM: Dulbecco’s modified Eagle’s medium
EE: Encapsulation efficiency
ECM: Extracellular matrix
ERK1/2: Extracellular signal-regulated kinase
FBS: Fetal bovine serum
FITC: Fluorescein isothiocyanate
GC–FID: Gas chromatography–flame ionization detector
G-MDSC: Granulocytic myeloid-derived suppressor cells
H&E: Hematoxylin/eosin
HPLC: High-performance liquid chromatography
iNOS: Inducible nitric oxide synthase
IBD: Inflammatory bowel disease
IPM: Inflammatory peritoneal macrophages
IFN-γ: Interferon γ
IL-4: Interleukin-4
LPS: Lipopolysaccharide
M-CSF: Macrophage colony-stimulating factor
MAPK: Mitogen-activated protein kinases
NF-κB: Nuclear factor-κB
PA: Patchouli alcohol
PI3K: Phosphatidylinositol-3 kinases
PLGA: Poly (lactic-co-glycolic acid)
PDI: Polydispersity index
PVA: Polyvinyl alcohol
Akt: Protein kinase B
ROS: Reactive oxygen species
qPCR: Real-time quantitative polymerase chain reaction
Tregs: Regulatory T cells
SV: Simvastatin
CMC-Na: Sodium carboxymethyl cellulose
TGF-β: Transforming growth factor-β
TEM: Transmission electron microscope
TNF-α: Tumor necrosis factor α
UC: Ulcerative colitis
XRD: X-ray diffractometer
