# Supplementary material for: “Two-birds-one-stone” colon-targeted nanomedicine treats ulcerative colitis via remodeling immune microenvironment and anti-fibrosis
Source: J Nanobiotechnology. 2022 Aug 30;20:389. doi: 10.1186/s12951-022-01598-0 (PMC9429315; doi:10.1186/s12951-022-01598-0)
Supplement: Supplementary file 1 — Additional file 1: Figure S1. Effect of PA on macrophage repolarization and synergistic effect with SV. (A–C) The mRNA levels of M1-associated pro-inflammatory cytokines (e.g., IL-1β, IL-6, and TNF-α) in PA-treated RAW264.7 macrophages, as measured by qPCR. (D, E) Western blot analysis of Akt/MAPK/NF-κB pathway-related biomarkers and M2-related MR expression after PA treatment. (F) The mRNA levels of the M1-related pro-inflammatory molecules (e.g., TNF-α, iNOS, and COX-2) and M2-related Arg1 in SV-treated RAW264.7 macrophages, as measured by qPCR. (G) IL-6 mRNA levels in LPS-induced peritoneal macrophages treated with PA (10 μM) and SV (0, 1, and 2 μM). Figure S2. Cytotoxicity study of PA and SV on (A) Caco-2 cells, (B) RAW264.7 cells, and (C) L929 cells. Cytotoxicity of the NPs in (D) L929 cells and (E) M2Φ. Figure S3. Anti-colitis treatment of synergistic drugs. (A) Schematic diagram of DSS-induced colitis and treatment. (B) Changes in daily bodyweight of each group during the trial period. (C) Statistical analysis and (D) images of colon lengths in each group (n = 4). Figure S4. Preliminary biosafety assessment of PA and SV. (A) Organ coefficients. (B) H&E staining of the major organs (Scale bar: 100 μm). Figure S5. Fluorescence images of (A) M1Φ and (B) L929 after incubation with the coumarin 6-labeled NPs (scale bar: 50 µm). (C, E) Histogram and (D, F) mean fluorescence intensity of the NPs-internalized M1Φ (LPS-induced RAW264.7 cells) and L929 cells were analyzed by flow cytometry (n = 3). Figure S6. Specific accumulation of CS-PLGA NPs in inflamed colons. Ex vivo imaging and radiant efficiency of (A, B) organs and (C) colons at 3 h. Ex vivo imaging and radiant efficiency of (D, E) organs and (F) colons at 5 h (n = 3). Figure S7. (A–E) Individual bodyweight curves and (F–J) DAI curves in CMC-Na, DSS, PA/SV, PLGA NPs, or CS-PLGA NPs groups (n = 6). Figure S8. Preliminary biosafety assessment. (A) Organ coefficients. (B) H&E staining of the major organs (Scale b [file 12951_2022_1598_MOESM1_ESM.pdf]

“Two-birds-one-stone” colon-targeted nanomedicine treats ulcerative colitis via remodeling immune microenvironment and anti-fibrosis

Jiaxin Zhang, Ante Ou, Xueping Tang, Rong Wang, Yujuan Fan, Yuefei Fang, Yuge Zhao, Pengfei Zhao, Dongying Chen, Bing Wang*, Yongzhuo Huang*

Additional figures and tables

Fig. S1 Effect of PA on macrophage repolarization and synergistic effect with SV. (A–C) The mRNA levels of M1-associated pro-inflammatory cytokines (e.g., IL-1β, IL-6, and TNF-α) in PA-treated RAW264.7 macrophages, as measured by qPCR. (D, E) Western blot analysis of Akt/MAPK/NF-κB pathway-related biomarkers and M2-related MR expression after PA treatment. (F) The mRNA levels of the M1-related pro-inflammatory molecules (e.g., TNF-α, iNOS, and COX-2) and M2-related Arg1 in SV-treated RAW264.7 macrophages, as measured by qPCR. (G) IL-6 mRNA levels in LPS-induced peritoneal macrophages treated with PA (10 μM) and SV (0, 1, and 2 μM).

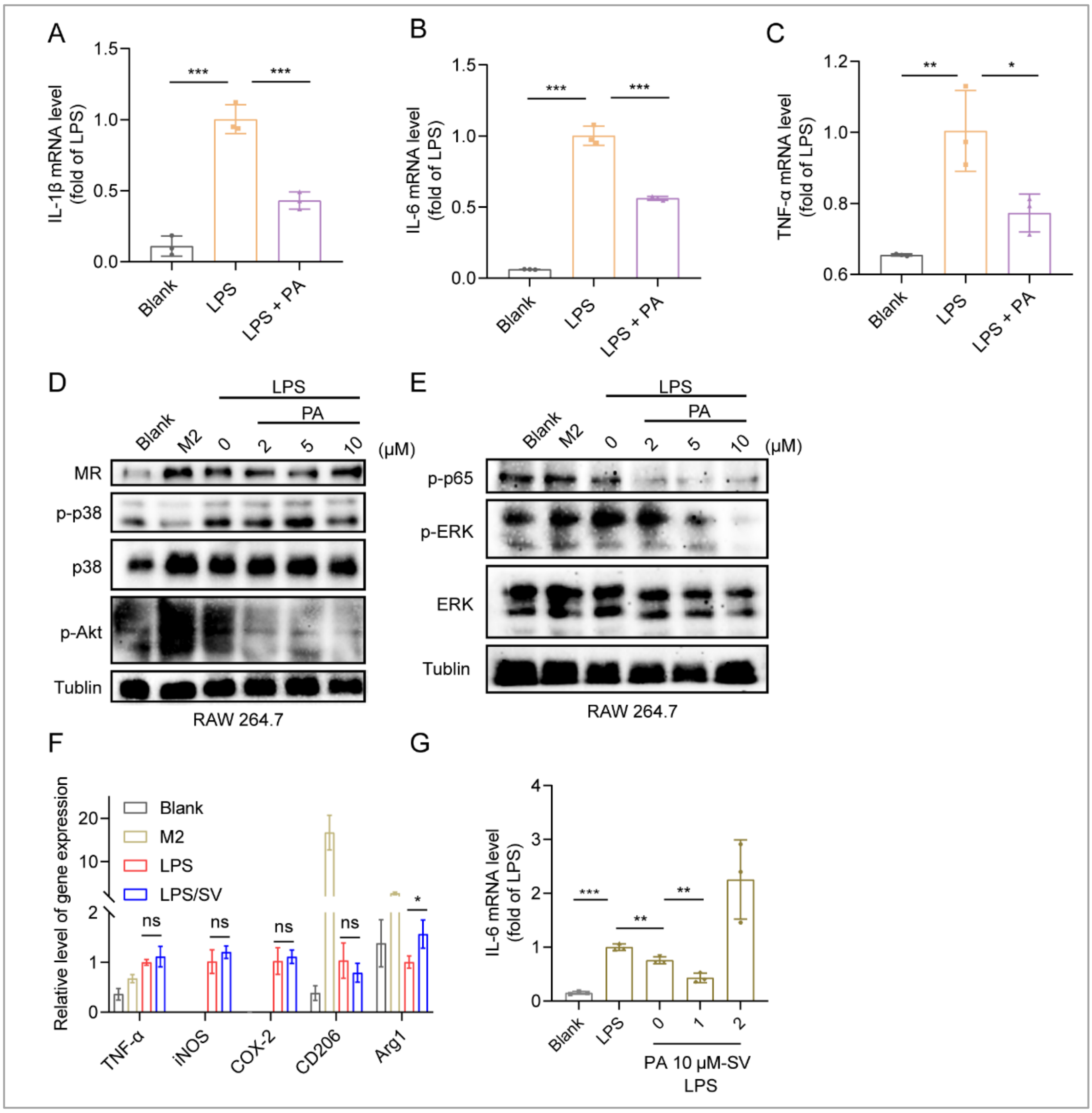

Fig. S2 Cytotoxicity study of PA and SV on (A) Caco-2 cells, (B) RAW264.7 cells, and (C) L929 cells. Cytotoxicity of the NPs in (D) L929 cells and (E) M2Φ.

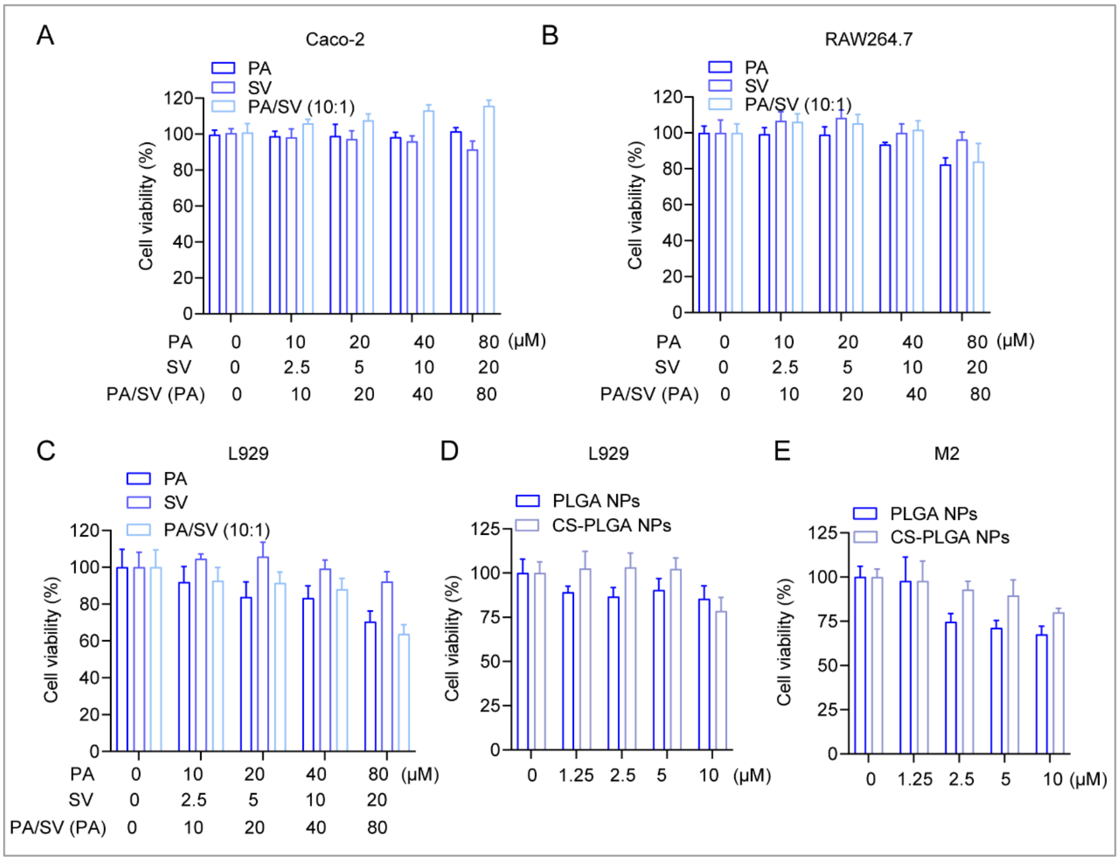

Fig. S3 Anti-colitis treatment of synergistic drugs. (A) Schematic diagram of DSS-induced colitis and treatment. (B) Changes in daily bodyweight of each group during the trial period. (C) Statistical analysis and (D) images of colon lengths in each group (n = 4).

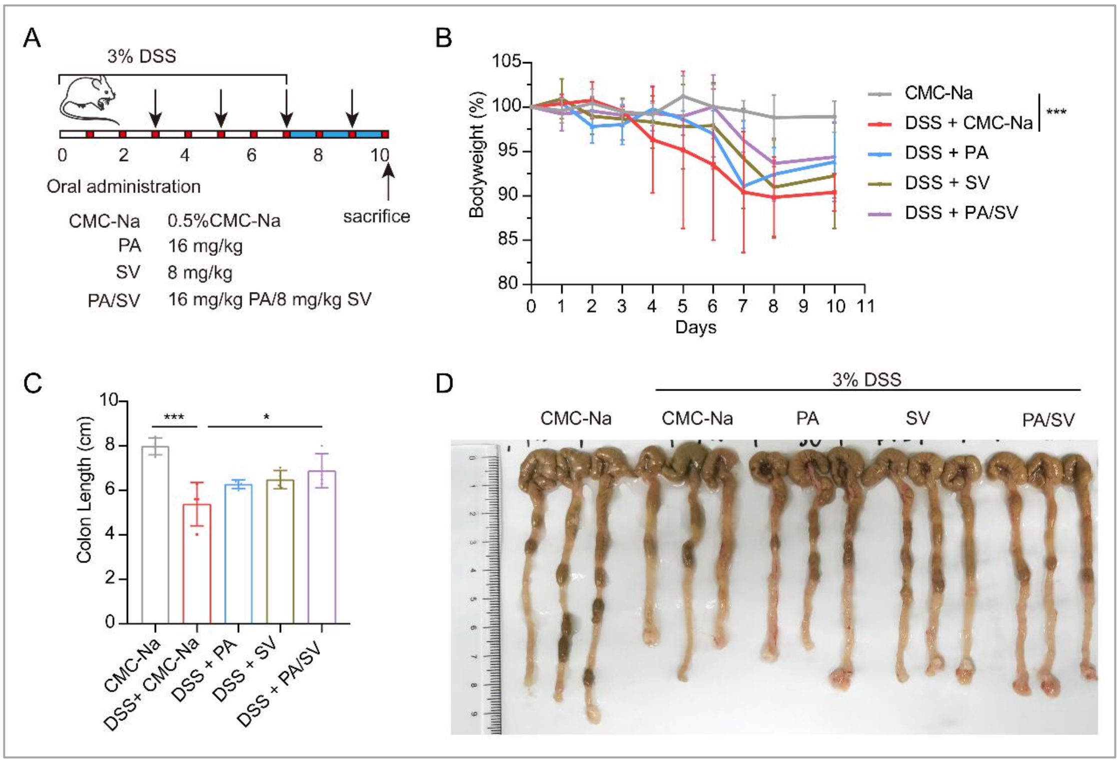

Fig. S4 Preliminary biosafety assessment of PA and SV. (A) Organ coefficients. (B) H&E staining of the major organs (Scale bar: 100 μm).

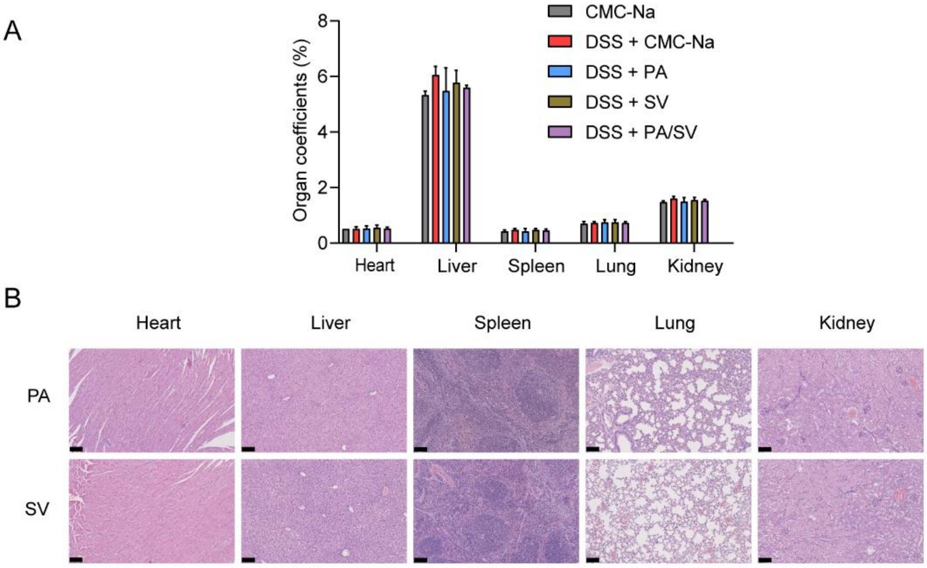

Fig. S5 Fluorescence images of (A) M1Φ and (B) L929 after incubation with the coumarin 6-labeled NPs (scale bar: 50 μm). (C, E) Histogram and (D, F) mean fluorescence intensity of the NPs-internalized M1Φ (LPS-induced RAW264.7 cells) and L929 cells were analyzed by flow cytometry, (n = 3).

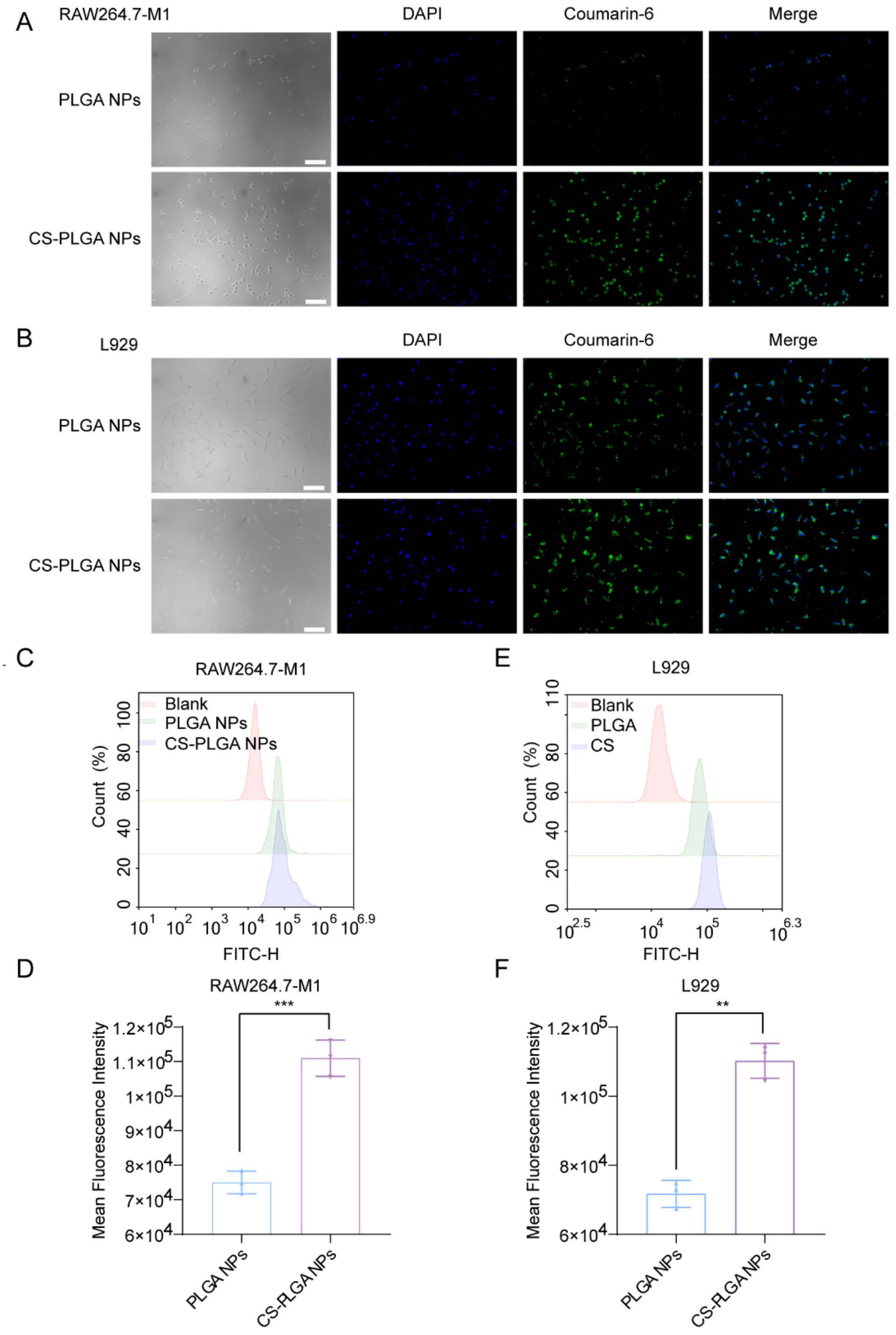

Fig. S6 Specific accumulation of CS-PLGA NPs in inflamed colons. *Ex vivo* imaging and radiant efficiency of (A, B) organs and (C) colons at 3 h. *Ex vivo* imaging and radiant efficiency of (D, E) organs and (F) colons at 5 h, (n = 3).

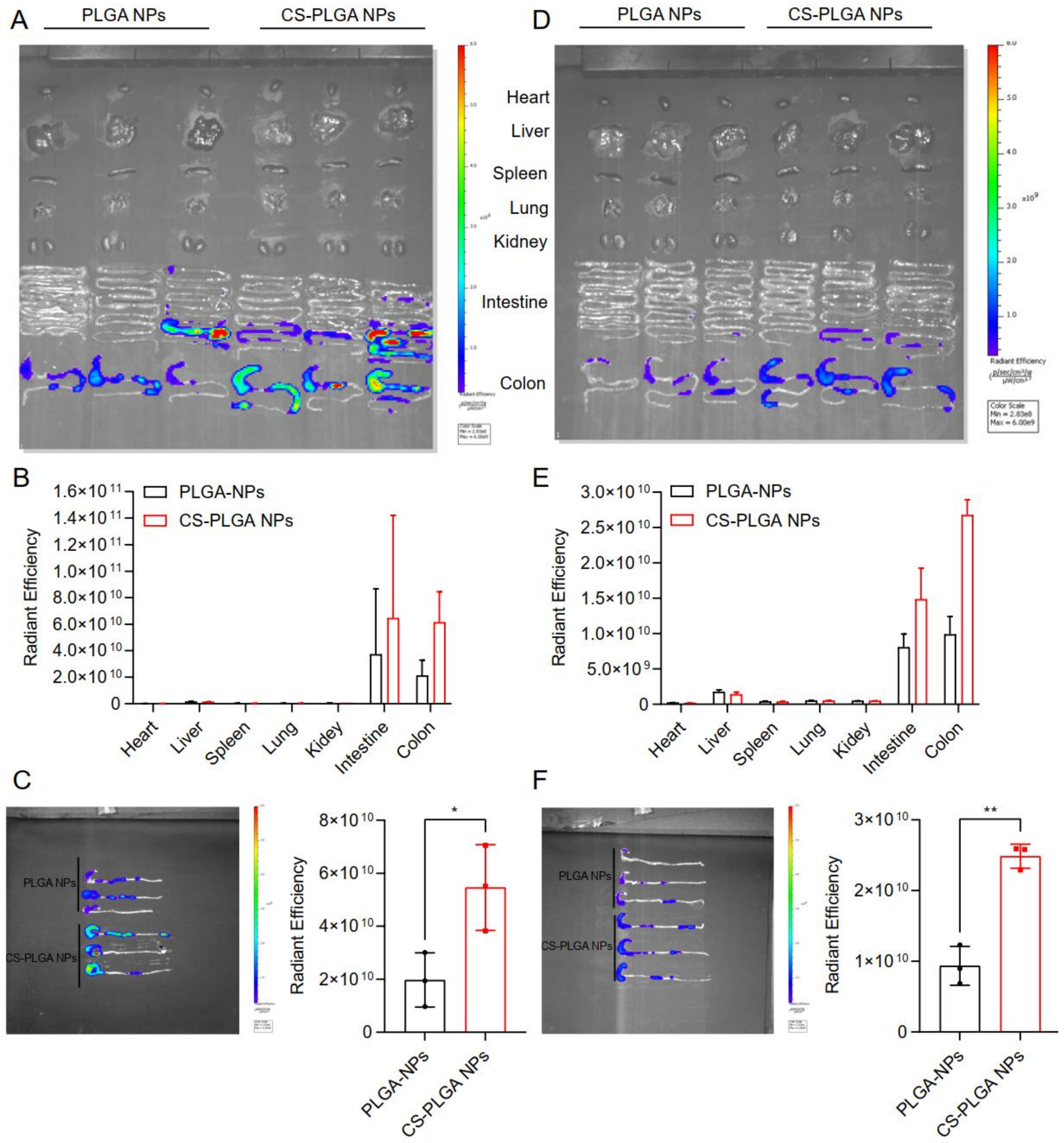

Fig. S7 (A–E) Individual bodyweight curves and (F–J) DAI curves in CMC-Na, DSS, PA/SV, PLGA NPs, or CS-PLGA NPs groups (n = 6).

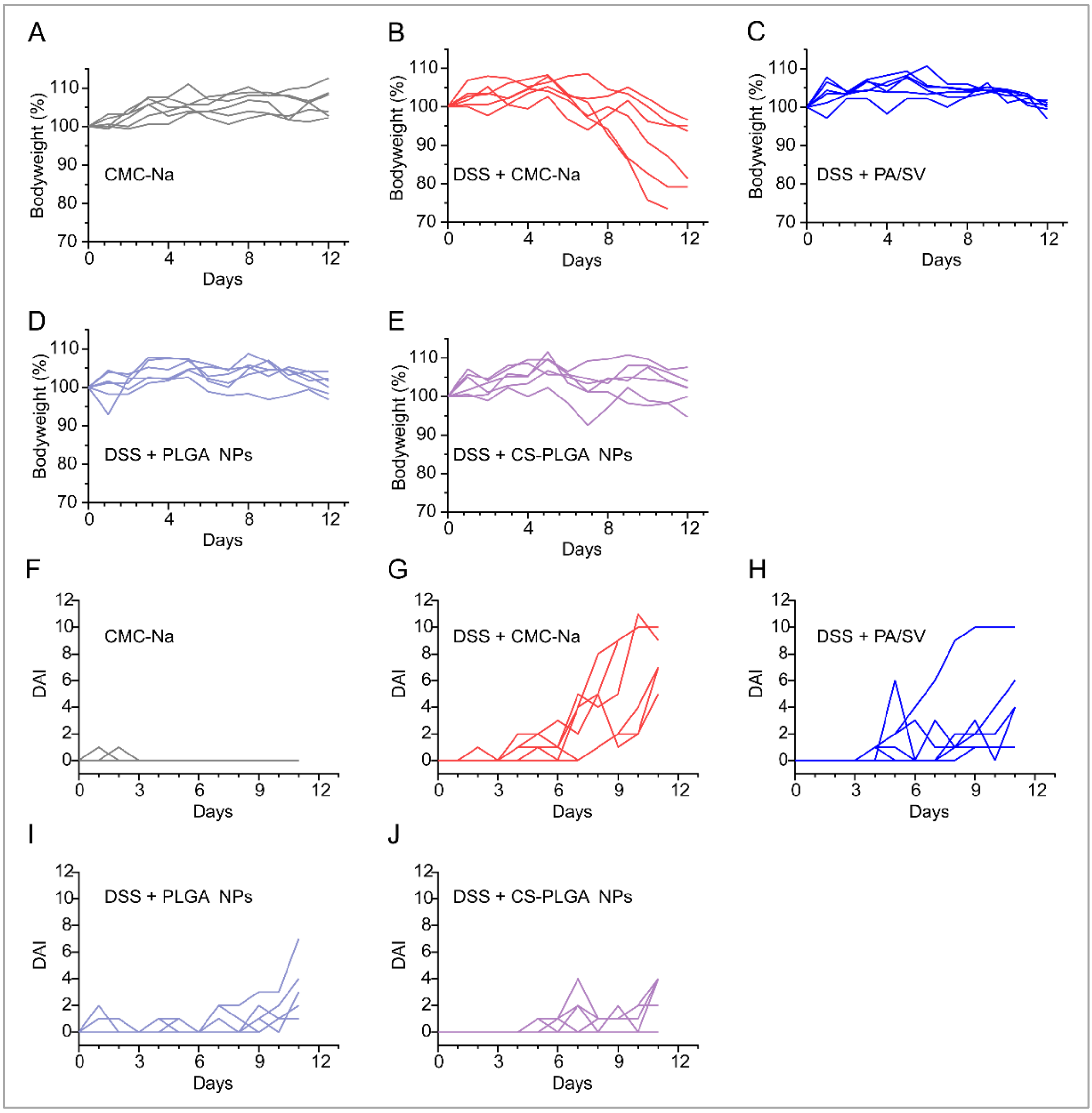

Fig. S8 Preliminary biosafety assessment. (A) Organ coefficients. (B) H&E staining of the major organs (Scale bar: 100 μm).

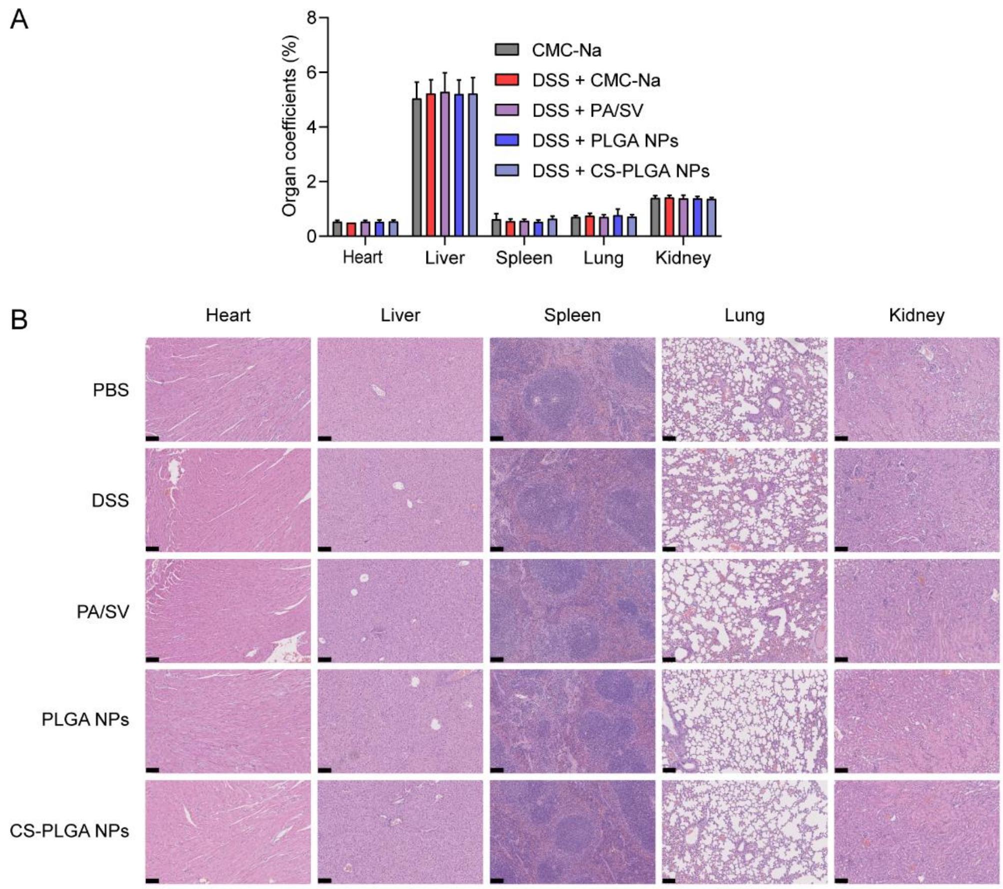

Fig. S9. The dot plots of M2Φ and DCs in the colon tissue.

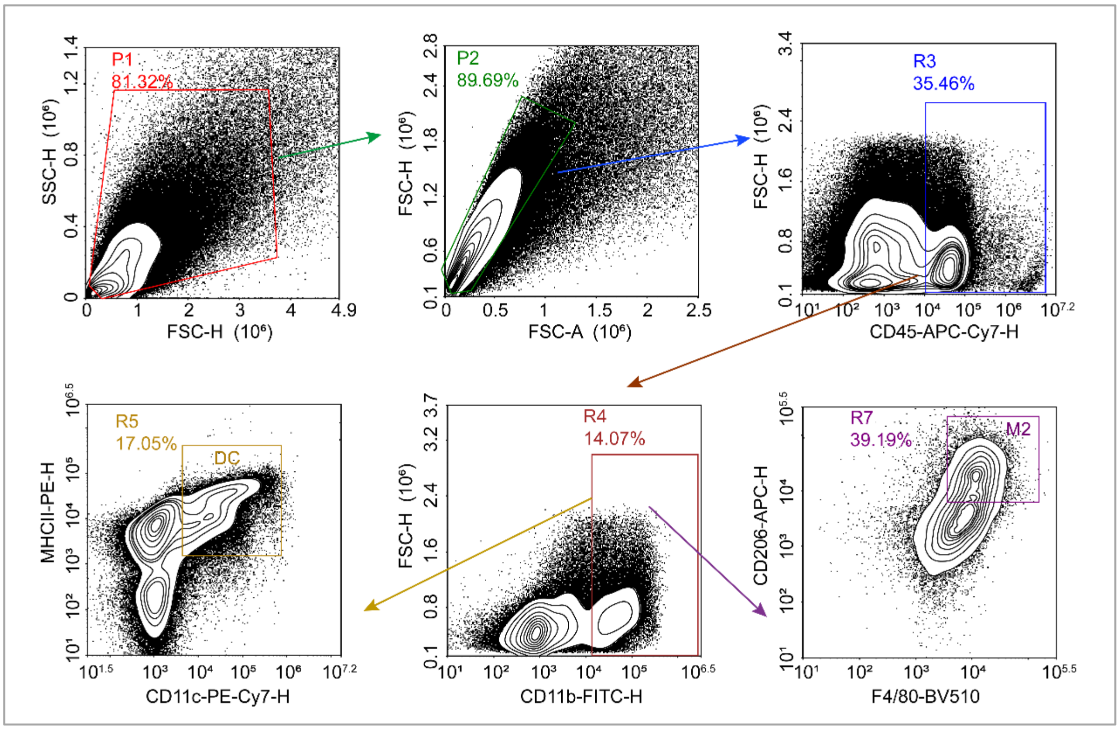

Fig. S10 The dot plots of neutrophils, inflammatory monocytes, and G-MDSCs in the colon tissue.

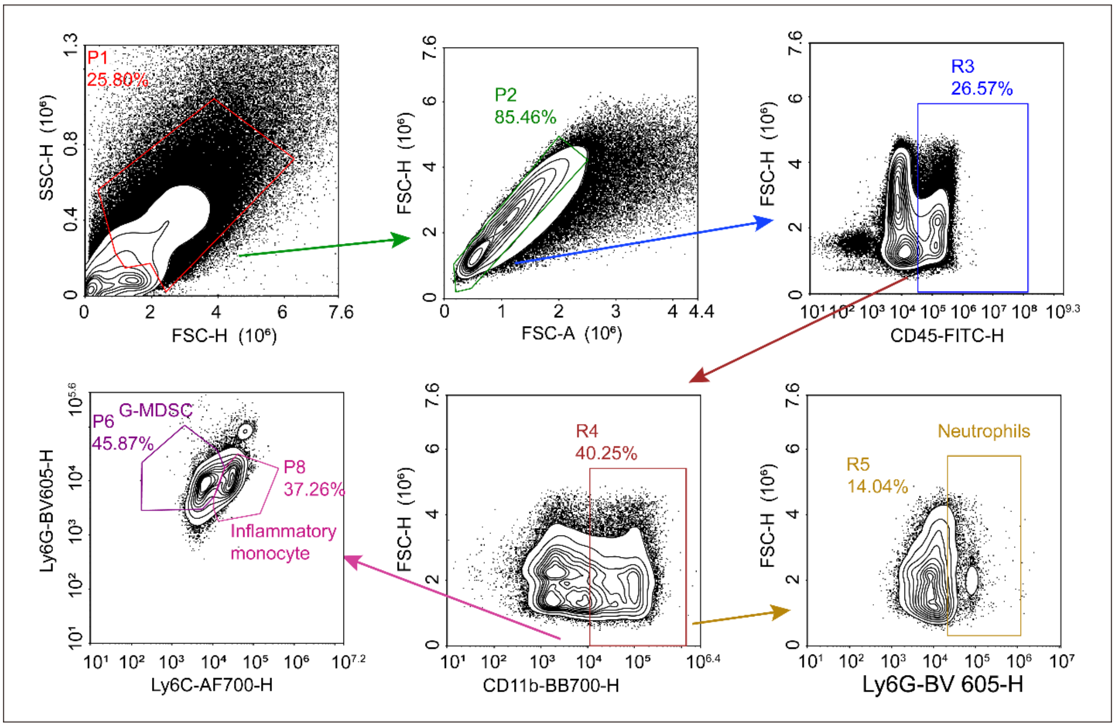

Fig. S11 The dot plots of Tregs in the colon tissue.

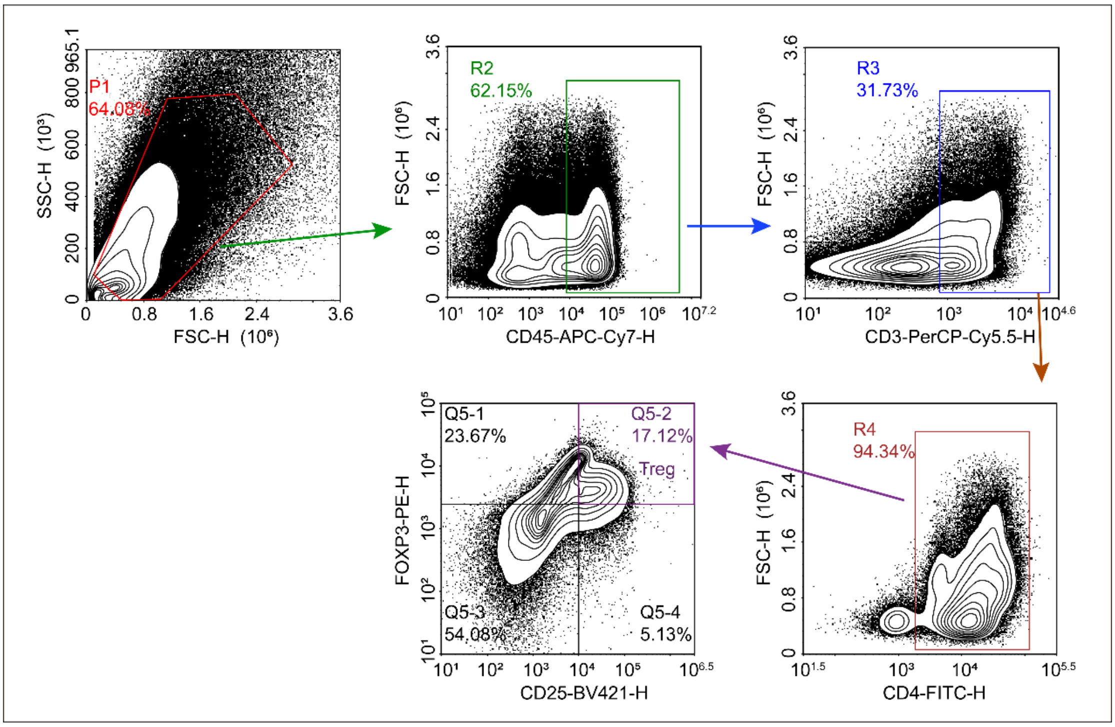

Table S1 The primer sequence used in qPCR

Gene	primer
TNF-α-F	CGAGTGACAAGCCTGTAGCCC
TNF-α-R	GTCTTTGAGATCCATGCCGTTG
IL-1β-F	CTTCAGGCAGGCAGTATCACTC
IL-1β-R	TGCAGTTGTCTAATGGGAACGT
IL-6-F	ACAACCACGGCCTTCCCTAC
IL-6-R	TCTCATTTCCACGATTTCCCAG
iNOS-F	ACATCGACCCGTCCACAGTAT
iNOS-R	CAGAGGGGTAGGCTTGTCTC
CD86-F	TTGTGTGTGTTCTGGAAACGGAG
CD86-R	AACTTAGAGGCTGTGTTGCTGGG
CD206-F	TCTTTGCCTTTCCCAGTCTCC
CD206-R	TGACACCCAGCGGAATTTC
Arg1-F	GAACACGGCAGTGGCTTTAAC
Arg1-R	TGCTTAGCTCTGTCTGCTTTGC
COX2-F	TGATCGAAGACTACGTGCAACA
COX2-R	AAAAGCAGCTCTGGGTCGAA
GAPDH-F	GGAAGGTGAAGGTCGGAGT
GAPDH-R	CCTGGAAGATGGTGATGGG
IFN-γ-F	AGCAACAGCAAGGCGAAA
IFN-γ-R	CTGGACCTGTGGGTTGTTGA
IL-12 F	AGACATCACACGGGACCAAAC
IL-12 R	CCAGGCAACTCTCGTTCTTGT

Table S2 Disease activity index (DAI) scoring

Scores	Weight loss（%）	Stool consistency	Stool bleeding
4	>15	watery diarrhea	-
3	11-15	very soft	visible blood traces in stool
2	6-10	-	-
1	1-5	slightly soft	hemoccult positive
0	none	well-formed pellets	no blood

Table S3 Characterization of the NPs

	PLGA-NPs	CS-PLGA NPs
Particle mean size (nm)	372.2 ± 30	351.6 ± 21
PDI	0.196 ± 0.037	0.202 ± 0.042
Zeta potential (mV)	-27.2 ± 4.0	9.7 ± 4.4

Table S4 Drug encapsulation efficiency and drug loading efficacy

	PLGA-NPs	CS-PLGANPs
EE (SV)%	65.3 ± 2.7	74.7 ± 6.3
EE (PA)%	40.2 ± 3.6	43.5 ± 0.5
DL (SV)%	1.3 ± 0.1	1.4 ± 0.3
DL (PA)%	2.08 ± 0.32	2.97 ± 0.78
